# The Trihelix transcription factor GT2-like 1 (GTL1) promotes salicylic acid metabolism, and regulates bacterial-triggered immunity

**DOI:** 10.1371/journal.pgen.1007708

**Published:** 2018-10-23

**Authors:** Ronny Völz, Soon-Kap Kim, Jianing Mi, Kiruthiga G. Mariappan, Xiujie Guo, Jean Bigeard, Santiago Alejandro, Delphine Pflieger, Naganand Rayapuram, Salim Al-Babili, Heribert Hirt

**Affiliations:** 1 Desert Agriculture, Division of Biological and Environmental Sciences and Engineering, King Abdullah University of Science and Technology, Thuwal, Saudi Arabia; 2 Institute of Plant Sciences Paris Saclay IPS2, CNRS, INRA, Université Paris-Sud, Université Evry, Université Paris-Saclay, Orsay, France; 3 Institute of Plant Sciences Paris-Saclay IPS2, Paris Diderot, Sorbonne Paris-Cité, Orsay, France; 4 Univ. Grenoble Alpes, CEA, Inserm, BIG-BGE, Grenoble, France; 5 CNRS, BIG-BGE, Grenoble, France; University of Copenhagen, DENMARK

## Abstract

The Trihelix Transcription factor GT2-like 1 (GTL1) was previously shown to be a key regulator of ploidy-dependent trichome growth and drought tolerance. Here, we report that GTL1 plays an important role in coordinating plant immunity. We show that *gtl1* mutants are compromised in the regulation of basal immunity, microbial pattern-triggered immunity (PTI) and effector-triggered RIN4-mediated immunity. Transcriptome analysis revealed that GTL1 positively regulates defense genes and inhibits factors that mediate growth and development. By performing hormonal measurements and chromatin-immunoprecipitation studies, we found GTL1 to coordinate genes involved in salicylic acid metabolism, transport and response. Interaction studies and comparative transcriptomics to known data sets revealed that GTL1 is part of the MPK4 pathway and regulates oppositely the expression of differentially expressed genes in *mpk4* plants. We introduced the *gtl1* mutation in the *mpk4* mutant and thereby partially suppressed its dwarfism and the high resistance against a bacterial invader. Our data show that GTL1 is part of the MPK4 pathway and acts as a positive regulator of bacterial-triggered immunity and SA homeostasis.

## Author summary

The trihelix-transcription factor GT-2-like 1 (GTL1) belongs to the seven genes containing GT-2 family of the plant-specific trihelix transcription factors. Previously, GTL1 was shown to be a key regulator of ploidy-dependent trichome growth and drought tolerance. In this report, we show that GTL1 is part of the MPK4-signaling cascade that coordinates immunity to virulent and avirulent *Pseudomonas syringae* strains. *gtl1* mutants are compromised in basal immunity, PTI and ETI. Comparative transcriptomics revealed a common set of differentially regulated genes in *gtl1* and *mpk4*. In this context, GTL1 positively regulates defense genes and inhibits factors that mediate growth and development. Salicylic acid measurements and Chromatin-Immunoprecipitation assays indicate that GTL1 directly binds and regulates genes involved in SA-biosynthesis, transport and response. The *mpk4/gtl1* double mutant is compromised in the resistance to *Pst AvrRPM1* and partially restored in the growth inhibition compared to *mpk4* single mutant. In summary, the reduced resistance of the double mutant indicates MPK4 as a negative regulator of GTL1-mediated *AvrRPM1*-triggered immunity.

## Introduction

Plants are faced with a constant threat of potential infections by a multiplicity of pathogenic microorganisms in their habitat. Pathogen-independent preformed physical borders like the cuticle, cell walls and wax coating represents the first line of plant defense to prevent pathogen invasion. Once the first boundary is breached, plants rely on their innate immune system to cope with different sorts of invaders and to initiate an adequate pathogen-counteracting defense response.

Plant innate immunity can be subdivided in two different recognition and response systems that relies either on the perception of pathogen-associated molecular patterns (PAMP) by plasma-membrane localized receptors (PRR) or on bacterial effectors injected in the plant cell and their recognition by intracellular receptors encoded by nucleotide binding domain leucine-rich repeat proteins (NLR-proteins) [1]. The perception of PAMPs, like FLAGELLIN22 (flg22), a 22 amino acid bacterial flagellum peptide, by the plasma membrane-localised receptor FLAGELLIN-INSENSITIV2 (FLS2) activates the mitogen-activated protein kinase (MAPK) cascades as part of the pattern-triggered immunity (PTI) [2]. Effector-triggered immunity (ETI) is activated by pathogen-derived “avirulence” (*avr*) effectors injected into the plant cell by the bacterial type III-secretion system in *Pseudomonas syringae cv tomato* (*Pst*). On susceptible (*r*) hosts, type III effectors can contribute to virulence, by interfering with plant immunity at the level of NPR1-dependent SA signaling [3] or the activation of the MAPKs MPK4 and 11 [4]. Some effectors are recognised by specific disease resistance (*R*) gene products leading to ETI. R and Avr proteins often co-localize within the plant cell [5]. The most common and widely distributed class of R proteins has a central nucleotide binding (NB) domain and C-terminal Leu-rich repeats (LRRs) [6]. The activation of NB-LRR proteins triggers a multitude of robust defense responses comprising biochemical and cellular events, like localized programmed cell death (hypersensitive response) and massive transcriptional reprogramming to restrict pathogen propagation [7]. Resistance to *Pseudomonas syringae* strains expressing either AvrB and AvrRpm1 [8, 9] is conferred by *Pseudomonas syringae pv maculicola 1* (*RPM1*), a CC-NB-LRR R protein, that is peripherally associated with the plasma membrane [10]. In this context, RPM1-Interacting Protein 4 (RIN4) acts as a vital defense regulator [11] and is targeted by several pathogen effectors, such as AvrRPM1, AvrRpt2, AvrPto and AvrPtoB [12]. AvrRPM1 triggers RIN4 phosphorylation [5] by RIN4-INTERACTING RECEPTOR-LIKE PROTEIN KINASE [13] to promote the defense repression mediated by RIN4. However, plants producing RPM1 R-protein detect RIN4 phosphorylation and initiate ETI [14]. AvrRpt2 is a Cys-protease that passes through self-activation and cleavage in order to cleave RIN4 at the plasma membrane [15]. RIN4 degradation imposed by AvrRpt2 is considered as a bacterial strategy to bypass AvrRpm1 induced ETI in the presence of RPM1 [16]. Recent studies have shown that SA signaling is an integral part of ETI and PTI [17, 18]. Plant innate immunity activates MAP kinase cascades typically involved in early and late immune responses. MAPK cascades consist of three sequentially activated kinase modules composed of a MAPK kinase kinase, a MAPK kinase and eventually a MAPK, thereby linking upstream signals to downstream targets. In Arabidopsis as well as throughout the plant kingdom, the MAPK orthologues of MPK3, MPK4 and MPK6 represent the final step in the transmission of PAMP signals to respective target proteins by phosphorylation [1, 19]. Although at least six PAMP-activated MAPKs have been reported to date [20, 21], but so far clear evidence for a role in defense only exists for MPK3, MPK4 and MPK6, all three of which are required for complete activation of defense genes [22]. MPK3 and MPK6 are both activated by MKK4 and MKK5, but their upstream MAP3K(s) have not yet been unambiguously identified [23]. In contrast, there is clear evidence that the MAPKKK MEKK1 activates the MAPKKs MKK1 and MKK2, which converge to activate MPK4 [24–27].

MPK3 and MPK6 regulate the expression of a number of pathways, including phytoalexins, indole glucosinolate and ethylene biosynthesis [28–30]. MPK4 positively regulates basal resistance against pathogens [31], and about 50% of flg22-induced genes require MPK4 [22]. On the other hand, *mekk1*, *mkk1 mkk2*, and *mpk4* mutant plants exhibit extreme dwarfism and autoimmune phenotypes such as spontaneous cell death and constitutive defense gene expression [24, 26, 31]. However, mutations in the NLR protein SUMM2 suppress these phenotypes [32], suggesting that SUMM2 monitors the integrity of the MEKK1-MKK1/2-MPK4 pathway [33, 34]. The *Pseudomonas syringae* pathogenic effector HopAI1, targets MPK4 to block its kinase activity and activates SUMM2-dependent defense response. In addition to SUMM2, SUMM1 is also required for activation of defense responses in *mekk1*, *mkk1 mkk2*, and *mpk4* mutant plants and encodes the MAPKKK MEKK2 [35]. MEKK2 functions upstream of SUMM2 as MEKK2 overexpression results in constitutive activation of defense responses in a SUMM2-dependent manner [35]. Recently, CALMODULIN-BINDING RECEPTOR-LIKE CYTOPLASMIC KINASE 3 (CRCK3) was identified as SUMM3. CRCK3 is directly interacting with SUMM2 and is required for the constitutive defense responses of *mekk1*, *mkk1 mkk2*, and *mpk4* mutant plants and suggested to function as the “guardee” or “decoy” of SUMM2 [32]. However, negative regulation of flg22-induced gene expression occurs through MPK4 phosphorylation of the transcriptional regulator ASR3 (ARABIDOPSIS SH4-RELATED3) [36] and complementation of *mpk4* mutants by a constitutively active MPK4 leads to enhanced pathogen susceptibility [37]. ASR3 belongs to a plant-specific trihelix transcription factor family and functions as a negative regulator of PTI. ASR3 suppresses a large subset of PAMP-induced genes via MPK4-mediated phosphorylation.

The trihelix-transcription factor GT-2-like 1 (GTL1) belongs to the seven genes containing GT-2 family of the plant-specific trihelix transcription factor family [38, 39]. Phylogenetic analysis of the GT-2 members shows that GT2, DF1 and GTL1 form a small clade while the other homologues are more distantly related [38–40]. A characteristic for five of the GT-2 members is the highly conserved N- and C-terminal trihelix DNA binding domain that generally binds to GT *cis* elements (GT1 box, 5‘-GGTTAA-3‘; GT2 box, 5‘-GGTAAT-3‘; GT3 box, 5‘-GGTAAA-3’) [41–43]. Topological comparisons identified a well-conserved intervening central helix region (alpha-helical coiled-coil domain) of around 70 amino acids of unknown function. Bioinformatic analysis of GTL1 identified a putative 9-amino acid transactivation motif which fully matches to the transactivation domain previously identified for eukaryotic and viral transcription factors [44]. The loss-of-function mutant *gtl1* shows large trichomes with increased levels of endoreduplication while the overexpression of *GTL1* is sufficient to arrest the endocycling and cell growth in trichome and other leaf epidermal cells [44, 45]. GTL1 actively terminates ploidy-dependent cell growth by the transcriptional repression of *CDH1/FZR/CCS52*, an activator of the anaphase-promoting complex/cyclosome (APC/C), and is considered as a critical molecular link between developmental programming and cell-size control. In this regard, *GTL1* is expressed during the post-branching stage of trichome development, and the protein is nuclear localised. However, the expression is not restricted to leaf hairs but also found in petals, expanding roots [44], leaves [38], in the abaxial epidermis and stomata [46]. Furthermore, *GTL1* is involved in the abiotic stress adaption. In this context, GTL1 was shown to regulate water use efficiency and drought tolerance by the modulation of stomatal density via the trans-repression of *STOMATAL DENSITY AND DISTRIBUTION1* (*SDD1*) expression [46].

In this report, we show that GTL1 is part of the MPK4-signaling cascade that coordinates PTI and ETI. Comparative transcriptomics revealed a common set of differentially regulated genes by GTL1 and MPK4. GTL1 positively regulates defense genes and inhibits factors that mediate growth and development. Hormone measurements and Chromatin-Immunoprecipitation assays indicate that GTL1 directly binds and regulates genes involved in SA biosynthesis and response. The analysis of the *mpk4/gtl1* double mutant suggests a genetic linkage of both factors, indicated by its compromised resistance to *Pst AvrRPM1* and the increased growth compared to *mpk4* single mutant, respectively.

## Results

### GTL1 associates with the MAPK MPK4 *in vitro* and *in vivo*

**Fig 1:**
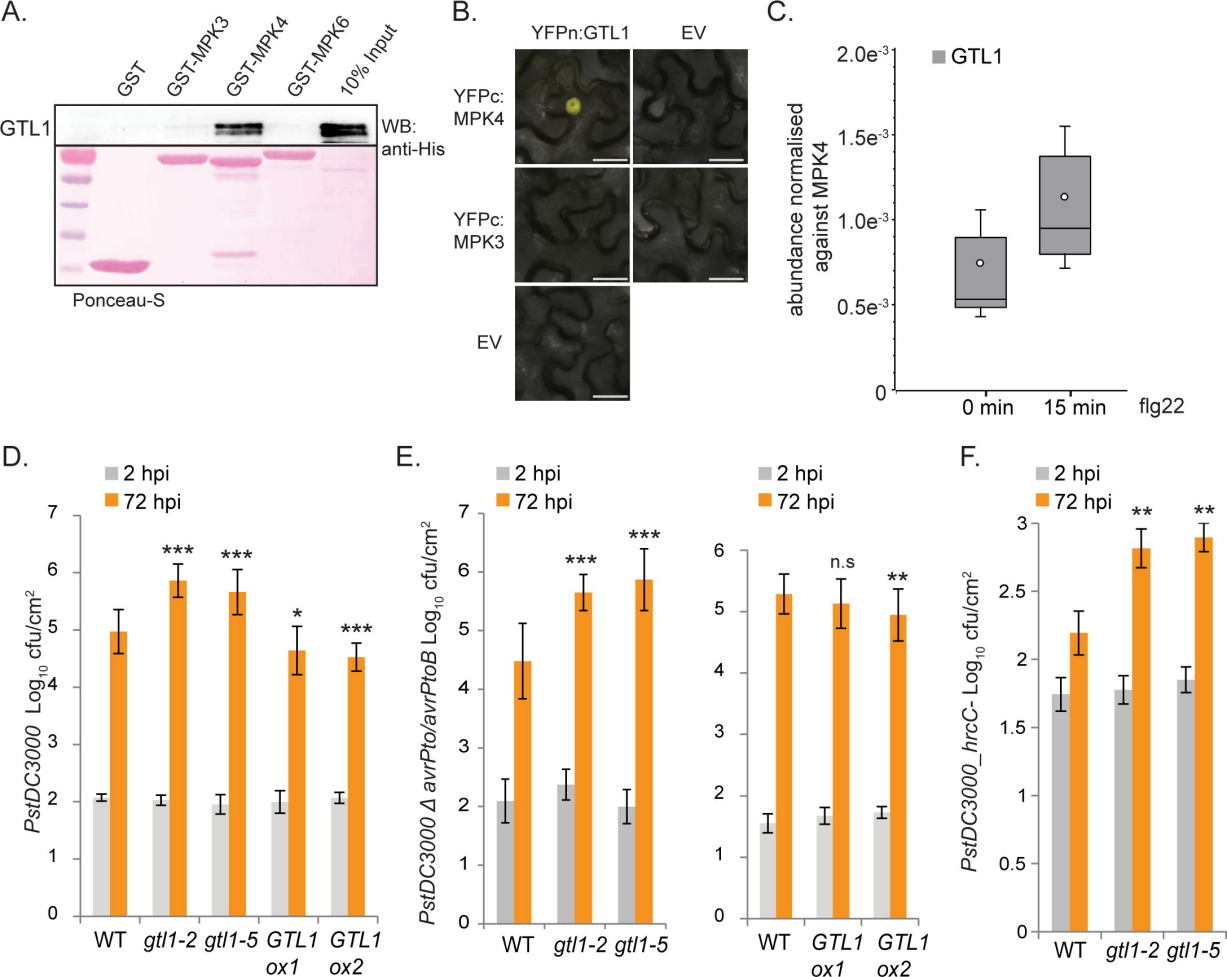
GTL1 associates with MPK4 and allelic *gtl1* mutants show higher susceptibility to various *Pseudomonas syringae* strains. **A)** *In-vitro* pull-down assays of MBPHis-tagged GTL1 show interaction with MPK4, unlike to MPK3, MPK6 and single GST. Pull-down assays were performed by incubating bacterial lysates of GST (lane 1), MPK3-GST (lane 2), MPK4-GST (lane 3), and MPK6-GST (lane 4) with GST beads followed by the incubation with bacterial lysate of MBPHis-tagged GTL1 (lane 6, 10% INPUT). The pull-downs and 10% INPUT were probed with an anti-His antibody (WB: anti-His). Proteins were stained with Ponceau-S. **B)** Nuclear interaction of GTL1 with MPK4. *Nicotiana benthamiana* epidermal cells were analyzed by Bimolecular Fluorescence Complementation. MPK3 and empty YFPn/ YFPc-vector (EV) serve as a negative control, scale bar = 25 μm. **C)** LC-MS/MS analysis of co-immunoprecipitated MPK4-GTL1 complex. A genomic TAP-tagged MPK4 construct was generated, stably introduced in *Arabidopsis thaliana*, and used for tandem affinity purification of MPK4 protein without treatment and 15 min after flg22 application. GTL1 was reproducibly identified via the GTL1-specific peptide EETLALLR whose relative abundance was normalized to MPK4 protein abundance. Box plots are depicted for GTL1 protein abundance associated with MPK4, boxes showing the interquartile range (IQR) 25^st^ to 75^th^ percentiles, inner ellipse representing the median, whiskers show the SEM. **D-F)** The allelic *GTL1* mutants *gtl1-2 and gtl1-5*, as well as two *GTL1*-overexpressing lines (*GTL1ox1*, *GTL1ox*) *were* challenged by the use of *PstDC3000*, *PstDC3000 Δ avrPto/avrPtoB* and *PstDC3000 hrcC-* **(*GTL1ox*, see under S2C Fig).** Plants, of three biological replicates (n = 30), were spray-inoculated with a bacterial suspension at OD_600_ 0.2, the density of colony-forming units (cfu) was analyzed 2 and 72 hours post inoculation (hpi). Error bars, mean ± SEM, statistical significance was analyzed by Student’s test, asterisks indicate significant differences compared to treated WT, * *p*≤ 0.05, ** *p*≤ 0.01, *** *p*≤ 0.001.

To identify unknown interaction partners of the immune MAP kinases MPK3, 4 and 6 that potentially contribute to immunity-associated processes in *Arabidopsis*, we analysed a collection of transcription factors (TF) following two stringent criteria. Firstly, these TFs were shown previously to function in abiotic stress adaptation, and secondly, *in silico* analysis by using the Eukaryotic Linear Motif resource [47] revealed putative MAP kinase docking sites. One of the transcription factors that emerged from this study was GTL1. The interactions of MAP kinases MPK3, 4 and 6 with GTL1 were assessed via *in vitro* pull-down assays by the use of MBP-His tagged GTL1 and GST-tagged MPK3, 4 and 6 (**Fig 1A**). Notably, we observed the predominate interaction of GTL1 with MPK4. However, we could not detect an association of GTL1 with MPK3 and MPK6, suggesting an exclusive biological function of GTL1 in the interplay with MPK4.

To evaluate the *in vitro* binding data, we applied two *in vivo* protein-protein interaction studies. Firstly, bimolecular-fluorescence complementation (BiFC) in *Nicotiana benthaniama* was performed by the use of GTL1-YFPn and MPK4-YFPc constructs. The interaction analysis showed a nuclear signal in tobacco epidermal cells demonstrating the interaction of MPK4 with GTL1 (**Fig 1B**). The negative control by using MPK3 and the empty-YFPc vector did not display a fluorescence signal.

To further evaluate the binding of MPK4 with GTL1, we performed a co-immuno-precipitation study coupled to mass spectrometry analysis. In this experiment, 18 day-old *Arabidopsis* plants were used that express an *MPK4*-Tandem Affinity Purification (TAP)-tagged genomic locus. This method bears the advantage to evaluate the interaction of 2 proteins at native protein levels and thereby minimising the risk to detect false-positive results imposed by the ectopic overexpression of the transgenes. We analysed three biological replicates in which GTL1 was identified and reproducibly quantified via the GTL1-specific peptide EETLALLR (amino acids 66 to 73) (**Fig 1C and S1 Table**), indicating that GTL1 interacts with MPK4 *in vivo*. In addition, the binding of MPK4 to GTL1 was evaluated in three biological replicates 15min after applying flg22. GTL1 was similarly reproducibly identified and the PAMP-treatment did not compromise the interaction of MPK4 with GTL1 (**Fig 1C**). In the LC-MS/MS analysis, we detected the GTL1-specific peptide EETLALLR with a significant Mascot score of 23.9 (**S1 Table**). By using the Eukaryotic Linear Motif resource, we performed a protein motif analysis of GTL1 which assigned the peptide EETLALLR to a MAP kinase docking domain at the N-terminus of the first trihelix-domain that comprises amino acids 62–71 (**S1A Fig**). The putative MAPkinase docking site RWPREETLAL in GTL1 follows the general MAPkinase docking pattern [KR]{0,2}[KR].{0,2}[KR].{2,4}[ILVM].[ILVF] with a valid probability of 4.324e^-03^ [47].

As part of the flg22-triggered signaling cascade, MPK4 commonly regulates target protein activity by phosphorylation on SP/TP sites in a PAMP-dependent manner. In several independent phosphoproteomic studies [48–52] SAAFEIAQS*PANR of GTL1 was found to be phosphorylated in a stress-dependent manner. Therefore, to discover motifs in GTL1 which are targeted for phosphorylation by MPK4, *in vitro* kinase assays were carried out by the use of the constitutively active version of MPK4. Surprisingly, despite the availability of 5 SP and 4 TP sites in GTL1 predominantly targeted by MPK4 [53], MPK4 did not phosphorylate GTL1 at any of the sites (**S1B Fig**). However, the results of the positive control *Target of Myb protein 1* (*TOM1*) were recently published by Rayapuram, et al. 2017 [54] which confirmed the functionality of the experimental setup. Taken together, we could not detect phosphorylation of GTL1 by MPK4 on SP or TP sites suggesting a regulation mechanism that relies on protein-protein interaction but not on phosphorylation.

### GTL1 and innate immunity

MPK4 and the associated signaling cascades are considered as key elements in *Arabidopsis* innate immunity. Thus, the interaction of GTL1 with MPK4 suggests that GTL1 might play a role in the defense in *Arabidopsis*. To test this hypothesis, pathogen assays were performed with different *Pseudomonas syringae DC3000* strains by the use of the allelic *GTL1* mutants *gtl1-2* (SALK_005965) [44] and *gtl1-5* (Salk_044308) [46], previously described as knock-out lines. In addition, we evaluated 2 independent *GTL1-GFP* lines (*GTL1ox1*, *GTL1ox2*) driven by the *UBIQUITIN10* promoter. The phenotype of *gtl1* is very similar to WT plants (**S2A Fig**) underpinned by a comparable leaf morphology and area, trichome number per leaf [44] and shoot dry weight [46]. However, the trichome and in particular the trichome-branch length is enlarged, and the stomatal density is reduced which in turn is accompanied by physiological characteristics like increased drought tolerance and increased water deficit tolerance [46]. The phenotype of the *GTL1ox* lines is indistinguishable from WT (**S2A Fig**) showing comparable biomass. For the pathogen application, we decided to apply spray inoculation of different *Pseudomonas* strains because this treatment reflects most closely the natural course of infection. To analyse the biological function of GTL1 in basal immunity, the allelic *gtl1* mutants and the two *GTL1ox* lines were treated by the use of the virulent hemibiotrophic pathogen *Pst DC3000* and *Pst DC3000 ΔavrPto/avrPtoB*. Two hours after spray infection, the infection levels in the different transgenic lines corresponded to those in WT plants indicating that stomatal immunity was not affected (**Fig 1D and 1E**). By contrast, after 72 hours, the allelic *gtl1* mutants showed a higher proliferation level of both *Pst DC3000* strains of approximately one log_10_ value than WT (**Fig 1D and 1E**). However, the bacterial titer in the *GTL1ox* lines was significantly reduced after *PstDC3000* infection compared to WT. This finding shows that *gtl1* mutants are compromised in basal resistance to *Pst* infection whereas the overexpression of *GTL1* leads to a reduced susceptibility. We evaluated these results and leaf-infiltrated *Pst DC3000* in WT plants and *gtl1* mutants. In accordance to the spray infection, the proliferation level of the bacteria was increased in the mutant background compared to WT (**S2B Fig**). Based on our study, we conclude that GTL1 functions as a positive regulator of basal immunity. We also tested the suseptibility of *gtl1* mutants to spray infection by the non-virulent PTI marker strain *Pst DC3000 hrcC-* which is mutated in the type-III secretion system and hence unable to deliver effector proteins. *gtl1* mutants showed higher proliferation levels of *Pst hrcC-* while the *GTL1ox* lines exhibit a WT-like resistance (**Fig 1F, S2C Fig**). These results indicate that GTL1 is a positive regulator of basal immunity and PTI.

### Basal hydrogen-peroxide level is affected in *gtl1* and *GTL1ox* lines

**Fig 2:**
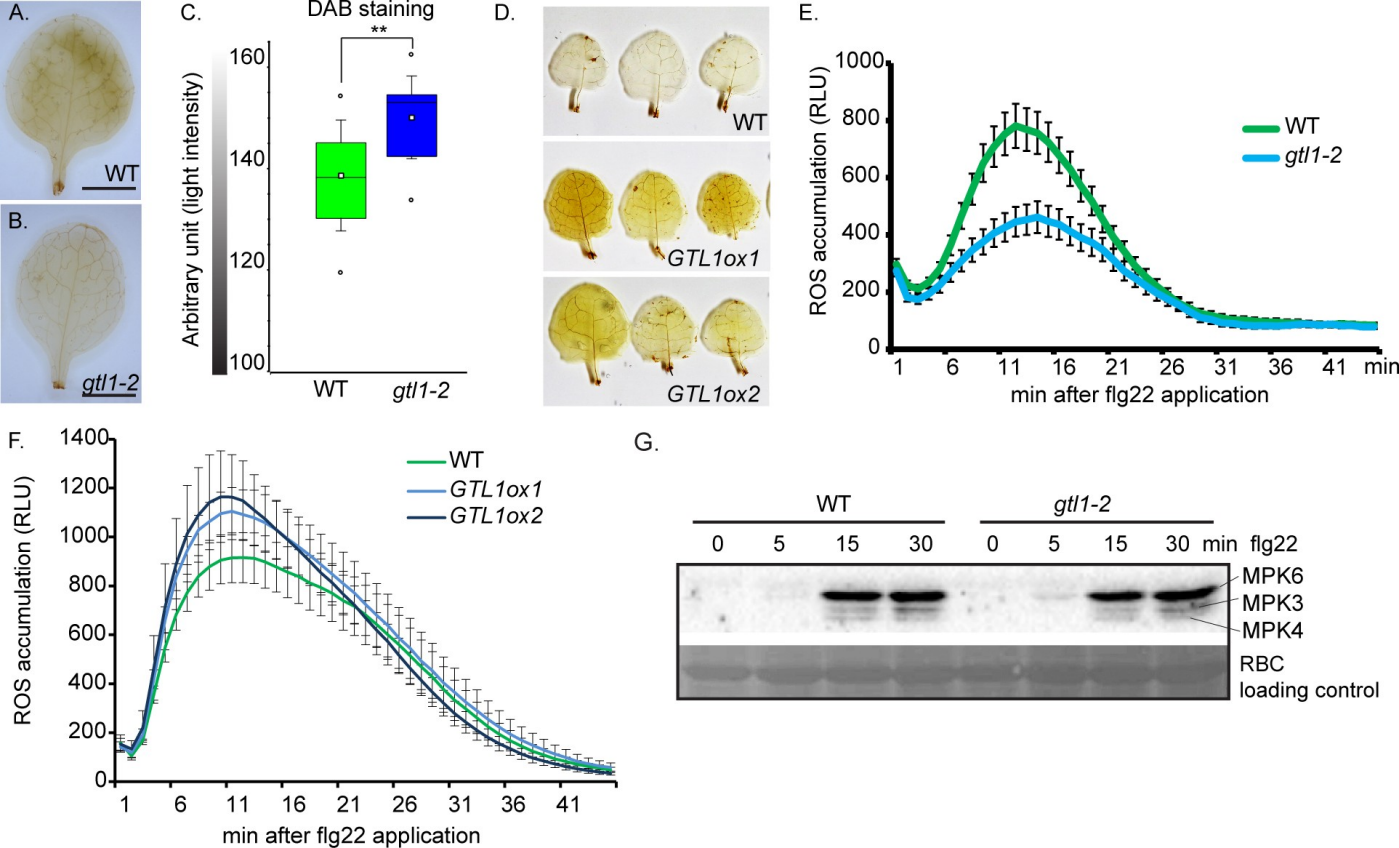
*gtl1* mutant shows reduced H_2_O_2_ levels in leaves and is compromised in flg22-triggered ROS-burst. **A-D**) Evaluation of *in-situ* H_2_O_2_ levels by 3,3'- diaminobenzidine staining (DAB) in untreated *gtl1-2* mutant, *GTL1ox1* and *GTL1ox2* compared to WT. Box plots are depicted for *gtl1-2* and WT, boxes showing the interquartile range (IQR) 25^st^ to 75^th^ percentiles, inner ellipse representing the median, whiskers show the SEM, outliers are depicted by dots (Min/Max range). Scale bar = 3 mm. Statistical significance was analyzed by Student’s test, asterisks indicate significant differences compared to WT, *p≤ 0.01. **E-F**) flg22-induced ROS burst assay of (**E**) *gtl1-2* compared WT plants and (**F**) *GTL1ox1* and *GTL1ox2* compared to WT plants,1μM flg22 treatment over 45 min, the data are shown as means ± SE from 36 leaf discs (3 biological replicates) of 5 week-old plants (negative control **S2D Fig**). **G**) flg22-induced MAPKinase-activation assays in *gtl1-2* and WT plants. 14 day-old seedlings were treated with 1 μM flg22 and samples were harvested at the indicated time points. Activation of MAPkinases 3, 4 and 6 were analyzed by immunoblot using pTpY-antibody recognizing the MAPKs in their activated form. Protein loading control was performed by Ponceau S staining for Rubisco (RBC).

To characterise the enhanced susceptibility of *gtl1* mutants and the increased resistance of *GTL1ox* lines in more detail, the levels of the reactive oxygen species (ROS) H_2_O_2_ was assessed by 3,3'-diaminobenzidine (DAB) staining in untreated WT plants, *gtl1-2* mutant and *GTL1ox* lines. We observed that the H_2_O_2_ level in *gtl1* is reduced in comparison to WT as depicted by a weaker staining intensity (**Fig 2A–2C**). In contrast, two independent *GTL1ox* lines displayed intense staining after the DAB exposure which demonstrates higher H_2_O_2_ levels than WT (**Fig 2D**). These findings show that the basal H_2_O_2_ level depends on the GTL1 function. Furthermore, ROS production and release are among the first defense reactions in response to pathogen perception. The ROS burst after flg22 treatment was significantly reduced in *gtl1* mutants to approximately 50%, 15 min after application (**Fig 2E, S2D Fig**). However, the *GTL1ox* lines showed elevated ROS release after flg22 treatment (**Fig 2F**). The differences in the ROS efflux after flg22 application in *gtl1* and *GTL1ox* lines might be a direct consequence of the affected basal H_2_O_2_ levels. The activation of the flg22-triggered signaling cascade was evaluated by pTpY antibody-based immunoprecipitation that targets the phosphorylated MAPK3, 4 and 6 versions. The highest activation of the three MAPK was achieved 15 min after flg22 treatment in both *gtl1-2* and WT plants (**Fig 2G**). The comparable activation of MPK3, 4 and 6 suggests a function of GTL1 downstream of the flg22-induced MAP kinase signaling cascades.

### Comparative transcriptome composition

**Fig 3:**
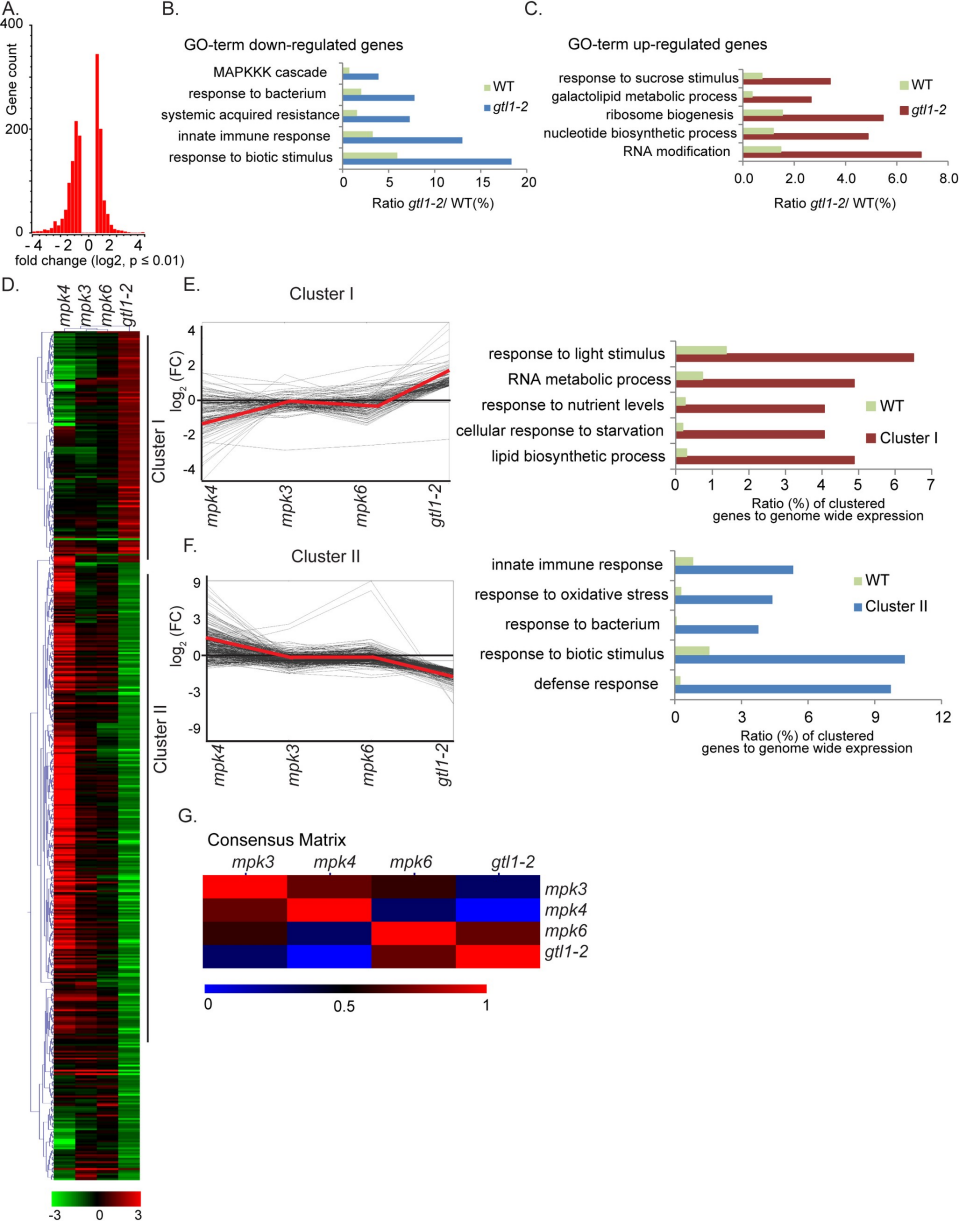
Comparative transcriptome analysis of *gtl1* and three immune MAPK mutants *mpk3*, -*4* and -*6*. **A)** Histogram of the Log_2_ distribution of up- and down-regulated genes in *gtl1-2* (**S1 Table**). **B-C)** Deregulated genes can be categorized in distinct Gene Ontology terms *gtl1-2* (**S1 Table**).**D)** Hierarchical clustering of *gtl1-2*, *mpk3*, *mpk4* and *mpk6* transcriptome highlights two main clusters showing opposite deregulated gene expression in *gtl1-2* and *mpk4*. log_2_ fold (p≤0.01, *gtl1-2*) of individual genes was used for clustering by using the average linkage method and Pearson Correlation (MeV4.0) (**S2 Table**).**E)** Centroid graph (red) and individual expression graphs dedicated to Cluster I. GO term analyses of genes grouped in Cluster I.**F)** Centroid graph (red) and individual expression graphs dedicated to Cluster II. GO term analyses of genes grouped in Cluster II.**G)** Consensus matric (Non-Negative Matrix Factorization, Cluster samples) shows most divergent gene expression between the total set of deregulated genes in *gtl1-2* and *mpk4*.

Firstly, to identify biological processes and genes that are governed by GTL1, the transcriptome of 14 day-old plants of *gtl1-2* mutant and WT was analysed by performing *RNA*-seq. At a stringency of *p*≤0.01, 1448 genes differently regulated genes (**S1 Table**) could be identified that show a log_2_ fold change from -5.29 to -0.53 of negatively regulated genes and from 0.52 to 5.00 of positively regulated genes. Among these 1448 genes, 678 genes are up-regulated, and 770 genes are down-regulated (**Fig 3A**). The GO term analysis of down-regulated genes revealed gene functions for Innate Immune Response, Systemic Acquired Resistance and Response to biotic stimulus and suggests a reduced ability of *gtl1-2* in these processes (**Fig 3B**). Furthermore, we found genes being down-regulated in *gtl1-2* that contribute to hydrogen peroxide metabolic process (**S1 Table**). For example, *ATRBOHC/RHD2* [55] and a substantial number of peroxidases (**S1 Table**) that contribute to H_2_O_2_ generation [56], such as *PRXCB*, *PER4*, *PRX37* and *PRX25*, are compromised in their expression. The GO terms in the set of up-regulated genes emphasised gene functions in Nucleotide Biosynthesis Process, Ribosome Biogenesis and Response to Sucrose Stimulus and can be summarized in support of plant growth and development (**Fig 3C, S1 Table**). All in all, the GO analysis indicates GTL1 as a positive regulator of immunity-related processes and a suppressor of biological functions related to plant growth.

Secondly, we sought to investigate whether the transcriptome composition of *gtl1* and *mpk4* is compromised in the same set of downstream targets. Therefore, deregulated genes in the *mpk* mutants [57] and *gtl1* were analysed by hierarchical clustering with mutants of MAPKs 3, 4 and 6 (**Fig 3D**). Interestingly, *mpk3* and *mpk6* mutants showed only a small overlap in gene expression with *gtl1*. However, a large number of genes in the *gtl1* mutant showed an opposite pattern of gene expression in the *mpk4* mutant. Among the two main clusters that were identified in the comparison between *gtl1* and *mpk4*, the 123 genes in cluster I are up-regulated in *gtl1-2* and down-regulated in *mpk4* (**Fig 3E, S2 Table**). The GO term analysis highlights gene functions in Response to Light, RNA Metabolism and Lipid Biosynthesis Process. Cluster II, comprising 319 genes, which are down-regulated in *gtl1-2* and up-regulated in *mpk4*, displays assigned GO terms for Innate Immunity and Response to bacterium (**Fig 3F, S2 Table**). The consensus matrix (**Fig 3G, S2 Table**) illustrates the dissimilarity of the gene sets in the *gtl1* and *mpk4* transcriptomes and also shows the difference to the transcriptomes of *mpk3* and *mpk6*. These findings indicate a genetic interaction of GTL1 and MPK4 in the regulation of distinct biological processes. To evaluate the *RNA*seq-based transcriptome comparison, the expression of three representative genes that contribute to SA-biosynthesis and response (**S2E Fig**) was analysed in the *gtl1* and *mpk4* mutant by qPCR. Firstly, *CAM-BINDING PROTEIN 60-LIKE G* (*CBP60g*) works cooperatively with *SARD1* [58, 59] to regulate the expression of *ICS1* to induce SA-metabolism; secondly, *PHYTOALEXIN DEFICIENT 3* (*PAD3*), that catalyses the conversion of dihydrocamalexic acid to camalexin [60, 61] and lastly, *ELICITOR-ACTIVATED GENE 3* (*ELI3-2/CAD8*) acting as alcohol:NADP+ oxidoreductase [62]. The expression of *CBP60g*, *PAD3 and CAD8* is diminished in *gtl1* when compared to WT, but enhanced in the *mpk4* mutant (**S3A–S3C Fig**).

### Salicylic acid metabolism and homeostasis are affected in *gtl1 and GTL1ox*

**Fig 4:**
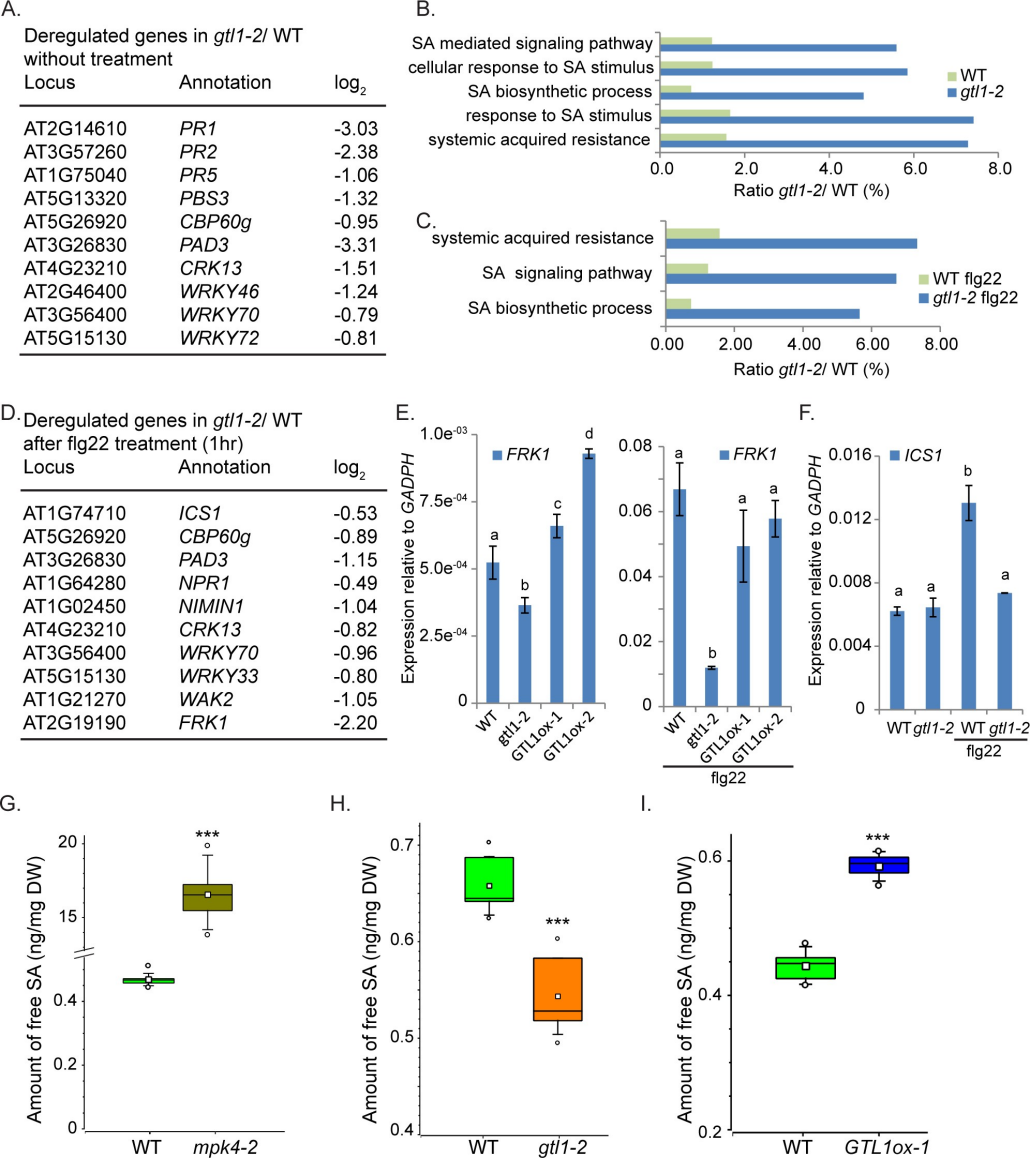
The expression of SA-metabolism/ signaling genes and the levels of salicylic acid are compromised in *gtl1-2* mutant. **A, D)** Depiction of down-regulated genes (**A**) in *gtl1-2* contributing to SA-biosynthesis and signaling without treatment (**S1 Table**) and (**D**) after flg22 treatment (**S3 Table**) compared to the respective expression in WT. **B, C)** Significantly down-regulated genes (p≤0.01, 769 genes) in *gtl1-2* before and after flg22 treatment (p≤0.0001, 715 genes) can be grouped in GO terms describing gene functions for SA-signaling and metabolism (**S2 and S3 Tables**).**E-F)** Expression of SA/PTI-response gene *FRK1* (**E, S3D Fig**) is reduced in *gtl1-2* before and after flg22 treatment but shows elevated expression in the *GTL1* overexpression lines in untreated conditions. The expression of the SA-biosynthesis gene *ICS1* (**F**) is diminished in *gtl1-2* after flg22 treatment. Statistical significance was analyzed by Student’s test Error bars, mean ± SEM, letters above bars represent significance groups, *p*≤ 0.01.**G-I)** Quantitative analysis of free salicylic acid by using LC-MS/MS. Box plots are depicted for (**G**) *mpk4-2*, (**H**) *gtl1-2* and (**I**) *GTL1ox1* compared to WT. Boxes showing the interquartile range (IQR) 25^st^ to 75^th^ percentiles, inner square representing the median, whiskers show the SEM, outliers are depicted by dots (Min/Max range). Statistical significance was analyzed by Student’s test. Asterisks indicate significant differences compared to WT,*** *p*≤ 0.001.

**Fig 5:**
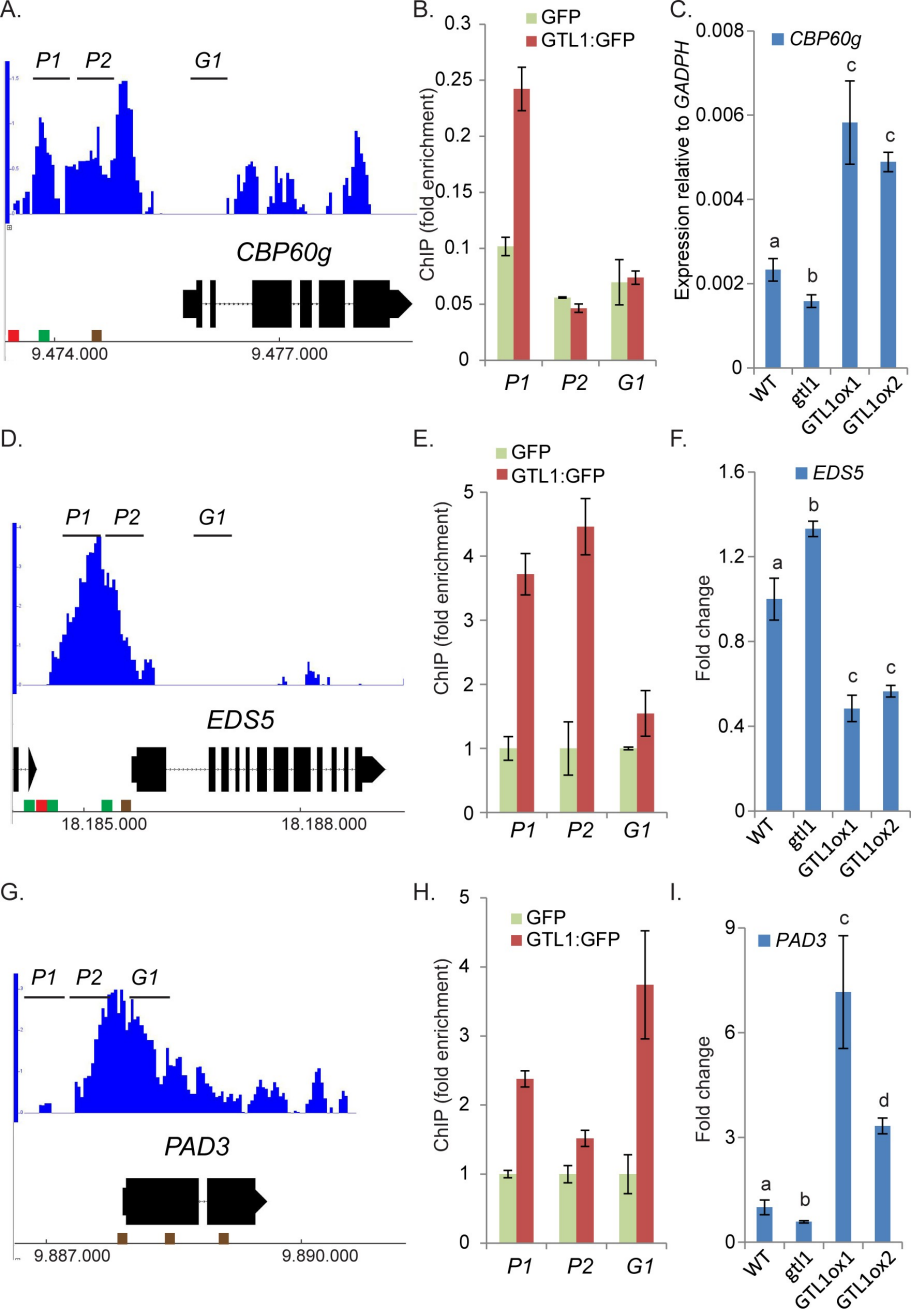
The SA-biosynthesis activator *CBP60g*, the SA-transporter *EDS5* and the SA-responsive gene *PAD3* are bound and regulated by GTL1. **A, D, G)** Microarray data, provided by Breuer et. al 2012 [45] show significant enrichment of GTL1 binding in the 5’-region of *CBP60g* (**A**), *EDS5* (**D**) and *PAD3* (**G**). Brown cube refer to the GT1-box, green cube indicates GT2-box and red cube shows GT3-box. **B, E, F)** ChIP-qPCR by using three biological replicates of *pUBI10*::*GTL1*:*GFP* expressing plants. GTL1 binding to genomic regions close to *CBP60g* (**B**), *EDS5* (**E**) *and PAD3* (**H**) were tested with sets of three primer pairs (*P1*, *P2*, *G1*) for each locus. Y-axis shows the fold enrichment in the *pUBI10*::*GTL1*:*GFP* lines normalized to GFP immunoprecipitation, driven by the *pUBI10* promoter (0.64 kb). **C, F, I)** The expression of *CBP60g* (**C**) and *PAD3* (**I**) is diminished in *gtl1-2* and elevated in *GTL1ox1* and *GTL1ox2*. The expression of *EDS5* is elevated in *gtl1-2* and reduced in *GTL1ox1* and *GTL1ox2*. Asterisks indicate significant differences compared to WT, * *p*≤ 0.05. ** *p*≤ 0.01. Statistical significance was analyzed by Student’s test. Letters above bars represent significance groups, *p*≤ 0.05.

In addition to *CBP60g*, the *RNA*seq-based transcriptome analysis also revealed that a number of genes are affected in *gtl1* mutants that contribute to the regulation of SA biosynthesis or its signaling events (**Fig 4A**). Compared to WT, the transcriptome analysis of *gtl1* mutants before and after PAMP application highlights in down-regulated genes GO categories for SA Biosynthesis, Systemic Acquired Resistance and Response to SA (**Fig 4B and 4C**). In untreated plants, the genes involved in SA biosynthesis, such as *CBP60g*, *PBS3* and *WRKY46* (**Fig 4A**) as well as SA signaling and PAMP-response targets, such as *WRKY72*, *PR1* and *FRK1* (**Fig 4A and 4E**) are down-regulated. To assess PAMP-triggered SA-metabolism and signaling in *gtl1-2*, the transcriptome composition was analysed 1 hour after flg22-treatment (**S3 Table**). In this regard, the expression of the key-SA biosynthesis gene *ICS1*, as well as its transcriptional activator *CBP60g*, is diminished (**Fig 4D and 4F**). Furthermore, the expression of the genes considered as central factors in SA signaling *NPR1* and *NIMIN1* is reduced (**Fig 4D)** as well as those of *FRK1* (**S3D Fig**), *WAK2* and *PAD3* contributing to SA-mediated response (**Fig 4D and 4E**). If GTL1 acts as an activator of genes involved in SA metabolism and signaling, then the expression of these genes is expected to be predominantly elevated in *GTL1*ox lines compared to the *gtl1* mutant and WT, respectively. Indeed, the expression *FRK1*, *CBP60g and PAD3* (**Figs 4E, 5C and 5I**) are significantly increased in the *GTL1*ox lines. These results indicate that GTL1 functions as a positive regulator of SA-mediated processes.

Consequently, we determined the levels of free SA in WT, *mpk4-2* and *gtl1-2* mutant, and *GTL1ox1* line in at least three biological replicates. Peterson et al, 2000 [31] showed that the SA accumulation in *mpk4* mutants is up to 10 fold higher than WT and our measurement are in accordance with these results (**Fig 4G**). After analysing 6 biological replicates, we determined a concentration of 16.56 ng SA /mg dry weight in *mpk4* compared to 0.47 ng/mg in WT. The high SA values in *mpk4* indicate MPK4 as a repressor of SA accumulation. Remarkably, the basal SA amount in the *gtl1-2* mutant is consistently lower than WT levels (**Fig 4H**), while the basal SA concentration in the *GTL1ox* line is significantly increased compared to WT (**Fig 4I**). Taken together, these results indicate that GTL1 is a positive regulator of genes involved in SA biosynthesis and promotes basal SA accumulation.

### GTL1 regulates genes involved in SA-metabolism and signaling

In genome-wide binding studies (ChIP-chip) [45], the association of GTL1 to regulatory sequences upstream and downstream of a large set of genes was revealed. A consensus binding motif for GTL1 was identified and described as GT3 box [5’-GGTAAA-3’]. In a previous *in-vitro* study, it was shown that the N-terminal DNA binding domain of GTL1 associates both to GT1 and GT2 boxes [46]. The ChIP-chip approach was carried out by the usage of the whole aerial part of 12 day-old *gtl1-1* plants that were complemented by *pGTL1*::*GTL1*:*GFP*. Among the total number of 2398 target genes, GTL1 was found to bind to the promoter regions of *CBP60g*, *EDS5* which codes for an SA-transporter [63] and *PAD3* (**Fig 5A, 5D and 5G**) as indicated in the Integrated Genome Browser diagram. To evaluate the association of GTL1 to these direct target genes, we performed Chromatin-Immunoprecipitation (ChIP) followed by quantitative PCR (qPCR) using gene-specific primer sets (P1, P1, G1). 14 day-old *Arabidopsis* seedlings expressing *pUBI10*::*GTL1*:*GFP* (**S4A Fig**) were generated and 3 independent transgenic lines were selected and used to confirm the binding of GTL1 to selected chromatin regions. To discriminate against false-positive binding caused by GFP, a negative control expressing GFP under the *UBI10* promoter was employed (**S4A Fig**). By ChIP-qPCR, the binding preference of GTL1 to the promoter region of *CBP60g* close to the transcriptional start sequence (TSS) could be confirmed (**Fig 5B**). Intriguingly, in our ChiP-qPCR study, GTL1 binds predominantly to the region-790 bp to 707 bp upstream of the TSS that contains one *GT2* motif previously described as the binding motif of GT transcription factors [43]. Accordingly, the binding of GTL1 to the region upstream of the TSS of *EDS5* could be confirmed to the 5’region P1 and P2 (**Fig 5D and 5E**) [45]. Upstream of the TSS of *EDS5* several *GT* boxes can be found facilitating the binding of GTL1. Moreover, GTL1 binds to the promoter region of *PAD3* upstream of the TSS and as well in the 5’-ORF (**Fig 5G and 5H)**. Notably, several GT-boxes (*GT1*) dedicated to GTL1 binding can be found indicating specific binding of GTL1 to the *PAD3* genomic region. Taken together, in accordance with the genome-wide binding studies of Breuer et al, 2012 and the presented ChIP evaluations, *CBP60g*, *EDS5* and *PAD3* could be confirmed as direct downstream targets of GTL1. To find out whether GTL1 exerts transcriptional control on *CBP60g*, *EDS5* and *PAD3*, we analysed their expression in the *gtl1* mutant and the *GTL1ox* lines. On the one hand, the expression of *CBP60g* and *PAD3* is reduced in the *gtl1* mutant and elevated in the *GTL1ox* lines (**Fig 5C and 5I**) under untreated conditions which suggest GTL1 as a transcriptional activator of these genes under non-stress conditions. After flg22-treatment, the expression of *CBP60g* and *PAD3* is also reduced in the *gtl1* mutant, but in the *GTL1ox* lines, the expression is ambiguous for *CBP60g* and WT-like in the case of *PAD3* (**S4B and S4D Fig**). On the other hand, the expression of *EDS5* is elevated in *gtl1* and reduced in the *GTL1ox* lines (**Fig 5F**) which implies GTL1 as a repressor of *EDS5* expression. After flg22-treatment, the expression of *EDS5* is not broadly perturbed from WT (**S4C Fig**). Our results suggest GTL1 as a transcriptional regulator of these genes involved in SA-biosynthesis, transport and response.

### GTL1 regulates immunity against the bacterial effectors AvrRpm1 and AvrRpt2

**Fig 6:**
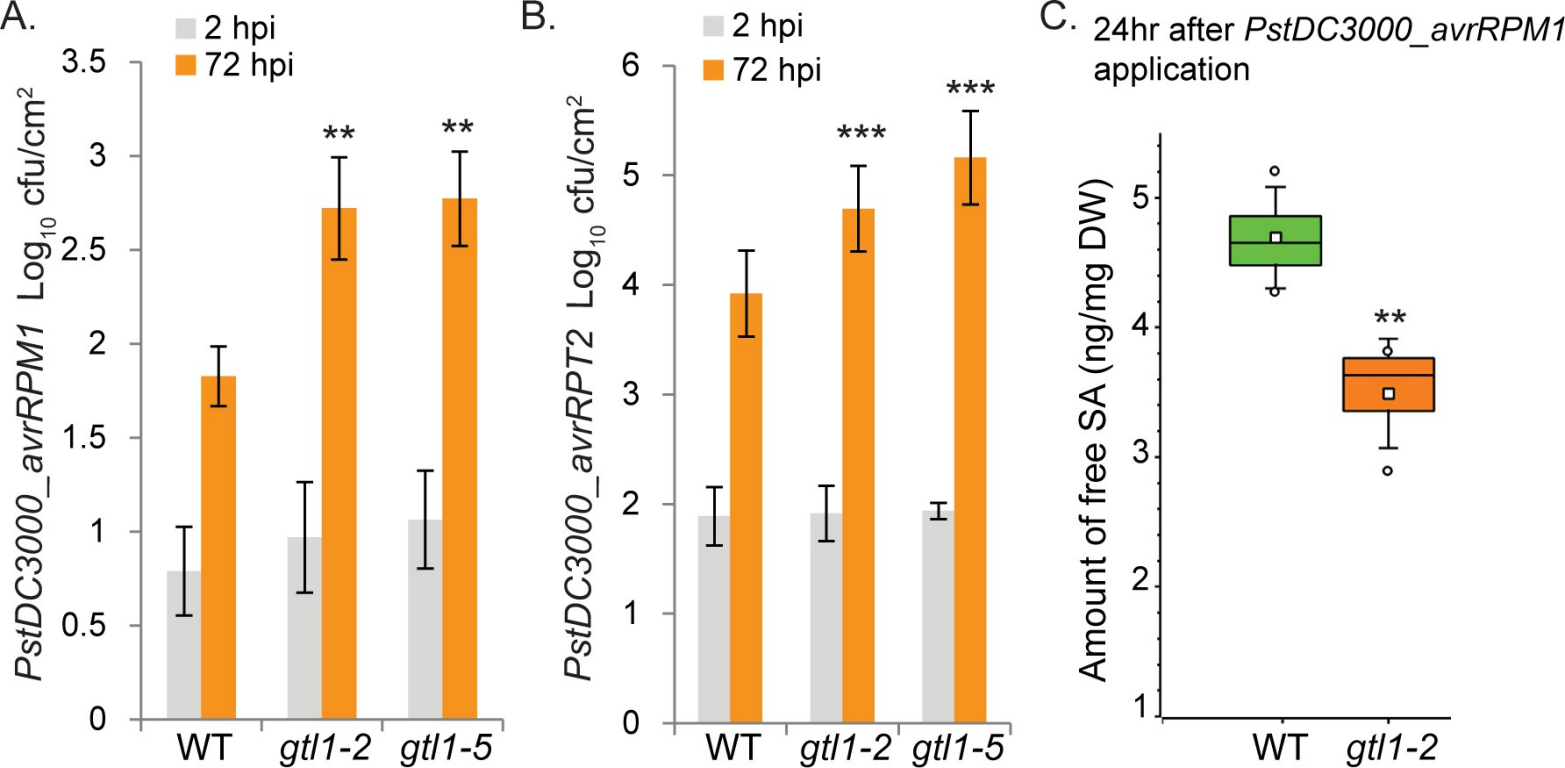
*gtl1* mutants are compromised in resistance to avirulent *PstDC3000* strains and accumulate less SA after *PstDC3000_avrRPM1* infection. **A-B)** The allelic *GTL1* mutants *gtl1-2 and gtl1-5 were* challenged by the use of *PstDC3000_avrRPM1 and PstDC3000_avrRPT2*. Results of two *GTL1*-overexpressing lines (*GTL1ox1*, *GTL1ox2*) are shown in **S Fig 4E and 4F**. Plants, of three biological replicates (n = 30), were spray-inoculated with a bacterial suspension at OD_600_ 0.2, the density of colony-forming units (cfu) was analyzed 2 and 72 hours post inoculation (hpi). Error bars, mean ± SEM, statistical significance was analyzed by Student’s test, asterisks indicate significant differences compared to treated WT, * *p*≤ 0.05 ** *p*≤ 0.01, *** *p*≤ 0.001. **C)** Quantitative analysis of free salicylic acid by using LC-MS/MS. Box plots are depicted for *gtl1-2* and WT, 24 hrs after *PstDC3000_avrRPM1* spray-inoculation, untreated conditions are shown in **Fig 4H**. Boxes showing the interquartile range (IQR) 25^st^ to 75^th^ percentiles, inner square representing the median, whiskers show the SEM, outliers are depicted by dots (Min/Max range). Statistical significance was analyzed by Student’s test. Asterisks indicate significant differences compared to WT,** *p*≤ 0.01.

Since MPK4 is also involved in effector-triggered immunity [37], we tested the bacterial strains *Pst DC3000_AvrRpm1* and *PstDC3000_AvrRpt2* which upon injection of the bacterial effectors trigger RIN4-dependent ETI [5]. Two hours after spray infection, the growth level of the bacteria in the transgenic lines was indistinguishable from WT (**Fig 6A and 6B**). However 72 hours after infection, we observed enhanced bacterial growth of about one fold change in either *Pst DC3000* strains in the allelic *gtl1* mutants (**Fig 6A and 6B**), while the *GTL1ox* lines are not broadly perturbed in their immunity compared to WT, respectively (**S4E and S4F Fig**). These results show that *gtl1* mutants are compromised in RIN4-*AvrRpm1*/*AvrRpt2* induced immunity which indicates a function of GTL1 in the effector-triggered immunity. To pinpoint whether GTL1 contribute to SA accumulation after bacterial infection, we determined the free SA levels by LC-MS/MS analysis 24 hours after *Pst DC3000_AvrRpm1* infection in the *gtl1* mutant. We found that the SA accumulation is significantly reduced to 3.5 ng/mg compared to WT showing an SA level of about 4.7 ng/mg in the average of 4 biological replicates, respectively (**Fig 6C**). Our results show that GTL1 is necessary for the SA accumulation as part of the ETI.

### *gtl1* partially restores susceptibility of *mpk4 and growth defects*

**Fig 7:**
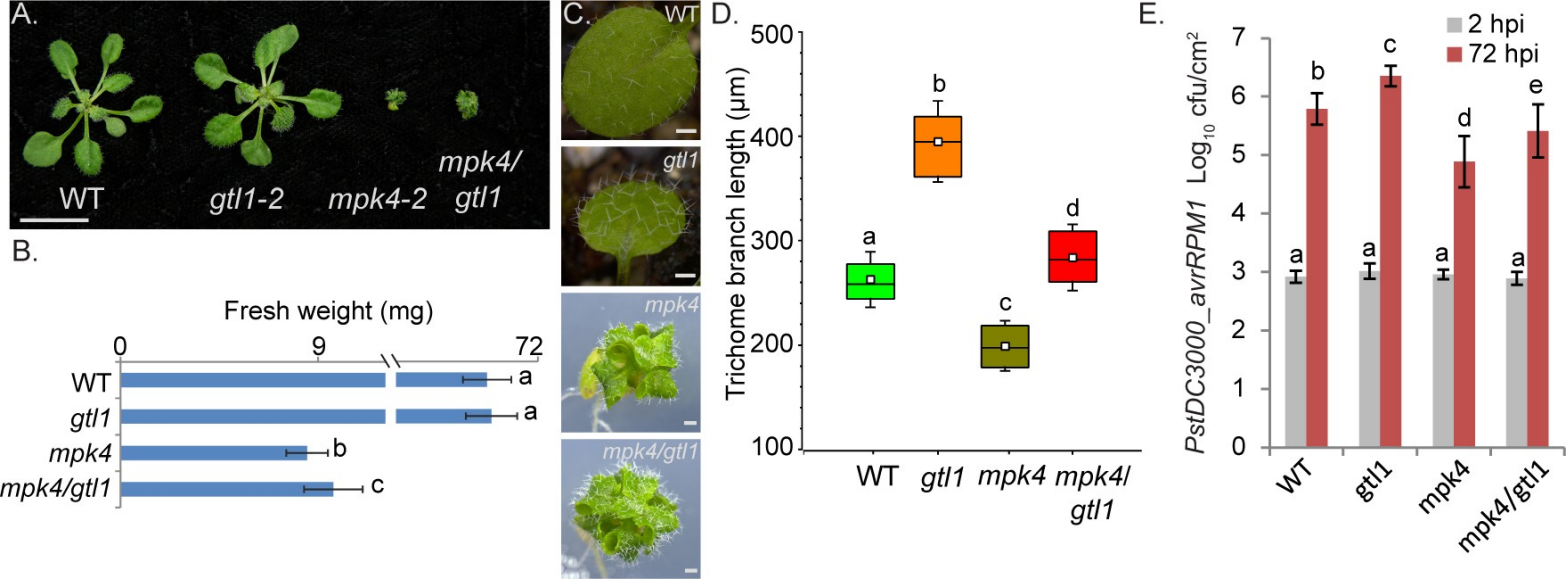
Mutation in *GTL1* partially restores the *mpk4* growth and resistance phenotype. **A-B)** Overview of the phenotype (**A**) and shoot fresh weight (**B**) of WT, *gtl1-2*, *mpk4-2* and *mpk4/gtl1* double mutants. The shoot fresh weight was analyzed of 4 week-old plants in 5 biological replicates and 7 week-old plants (**S4G Fig**). Error bars, mean ± SEM, statistical significance was analyzed by Student’s test. Letters above bars represent significance groups, *p*≤ 0.005. Scale bar = 1cm. **C-D)** Bright-field microscopy of WT, *gtl1*, *mpk4* and *mpk4/gtl1* leaves/plants. Scale bar = 500 μm. Error bars, mean ± SEM, statistical significance was analyzed by Student’s test. Letters above bars represent significance groups, *p*≤ 0.01. **D)** Quantitative analysis of trichome branch length of WT, *gtl1*, *mpk4* and *mpk4/gtl1* plants. Trichomes of 4 week-old plants were measured by the use of ZEN lite 2012 software. Boxes showing the interquartile range (IQR) 25^st^ to 75^th^ percentiles, inner square representing the median, whiskers show the SEM, Statistical significance was analyzed by Student’s test. Letters above boxes represent significance groups, *p*≤ 0.01. **E)** WT, *gtl1-2*, *mpk4-2* and *mpk4/gtl1* mutant were treated with *PstDC3000 avrRPM1* Plants, of three biological replicates (n = 30), were spray-inoculated with a bacterial suspension at OD_600_ 0.2, the density of colony-forming units (cfu) was analyzed 2 and 72 hours post inoculation (hpi). Error bars, mean ± SEM, statistical significance was analyzed by Student’s test. Letters above bars represent significance groups, *p*≤ 0.01.

Previously, it was reported that the strong autoimmune phenotype in *mpk4-3* largely depends on SUMM2 [34]. However, the accumulation of H_2_O_2_ and *PR* gene expression are only to some extent diminished in the *mpk4/summ2* double mutant and show still significant enhancement compared to WT [34]. Furthermore, the severe dwarfism of the *mpk4* mutant is not fully restored by the introduction of different allelic *summ2* mutations [34]. Eventually, these previous results indicate that MPK4 is involved in immune and growth regulation independently of SUMM2 [34]. To test whether defense response to bacterial attack in GTL1 depends on MPK4, we generated and analysed the *mpk4-2/gtl1-2* double mutant. As depicted in **Fig 7A and 7B**, the fresh weight of the double mutant is increased by about 14% of 4 week-old plants and 34% of 7 week-old plants (**S4G Fig**) compared to *mpk4-2* single mutants, respectively. Furthermore, the trichome branch length is extended in the double mutant compared to *mpk4* single mutant suggesting a partial suppression of developmental defects in *mpk4* plants by the *gtl1* mutation (**Fig 7C and 7D**). To evaluate the genetic interaction of MPK4 with GTL1 in the effector-triggered immunity, the double mutant was treated by *Pst DC3000 AvrRPM1*. At two hours after infection, the bacterial titer was indistinguishable from WT in *gtl1*, *mpk4* single mutants, and *mpk4/gtl1* double mutant thereby suggesting that stomatal immunity is not perturbed (**Fig 7E**). However, after 72 hours, the proliferation level in the *mpk4* single mutant was significantly reduced compared to WT, while the bacterial titer was elevated in *mpk4/gtl1* compared to *mpk4* single mutants (**Fig 7E**). Based on our findings, we postulate that MPK4 functions as a negative regulator of GTL1 in *AvrRPM1*-triggered RIN4-mediated immunity.

## Discussion

### Role of GTL1 in basal resistance and PTI

In this study, we identified the trihelix transcription factor GTL1 as a regulator of immunity. Using pathogen assays with virulent *Pst DC3000*, *Pst DC3000 ΔavrPto/avrPtoB* and non-virulent *Pst DC3000 hrcC-* strains, we showed that *GTL1* is a positive regulator of basal defense and PTI, respectively. Transcriptome analysis suggested that GTL1 functions on similar targets as the MPK4 pathway. However, whereas MPK4 negatively regulates the overlapping set of targets contributing to defense and immunity, GTL1 regulates them in a positive manner. In this context, the *mpk4* mutant exhibits enhanced resistance to *Pst DC3000* and elevated expression of defense markers [31]. Since PAMP-triggered MAPK activation is not affected in *gtl1* mutants, it is likely that GTL1 functions downstream of the MEKK1-MKK1/2-MPK4 cascade. This hypothesis is supported by the fact that GTL1 forms part of the MPK4 protein complex. We also found that GTL1 can directly interact with MPK4 and that this interaction is specific as no interaction was detected with the related immune MAPKs MPK3 and MPK6. Interestingly, we could not detect phosphorylation of GTL1 by MPK4, suggesting a regulation mechanism that relies on protein-protein interaction rather than phosphorylation.

### GTL1 is a positive regulator of SA biosynthesis and signaling but negatively regulates growth

The transcriptome pattern of *gtl1* mutants revealed that GTL1 is a positive factor for defense gene expression but a negative regulator of genes involved in growth, supporting the concept that growth and defense are inversely coupled. In agreement with a role of GTL1 in suppressing growth, *gtl1* mutants are also slightly bigger than WT plants under water-deficiency [46], but as shown here, this feature comes with the caveat of being more susceptible to pathogen attack. Conversely, *mpk4* mutant plants are dwarfed but are incredibly pathogen resistant. A characteristic feature of *mpk4* mutants is the increased SA level that correlates with its enhanced resistance to the virulent strains of *Pseudomonas syringae* [31]. Interestingly, SA amounts in *gtl1-2* mutant and lines expressing a constitutively active MPK4 version [37] were consistently lower than WT suggesting an opposing regulation of SA homeostasis. Since *gtl1* has reduced whereas *mpk4* massively, enhanced SA levels, one might be tempted to conclude that these different sensitivities could be solely due to SA amounts. However, this assumption is probably too simple as suppression of SA levels in *mpk4* mutants could only relieve the dwarf phenotype to some extent [31].

Transcriptome analysis confirmed a role of GTL1 as a positive regulator of SA and defense as well, by showing reduced levels of the SA/PAMP-marker genes, such as *PR1* and *FRK1*. This effect seems to be mediated both at the level of SA signaling genes, exemplified by *NPR1* and *NIMIN1*, as well as at the level of the biosynthesis gene *ICS1/SID2* and its regulator *CBP60g*. The analysis of available ChIP-chip data [45] indicated that GTL1 binds to a number of its target genes via interaction of the GT boxes. By ChIP-qPCR, we could verify that GT elements are involved in the regulation of the *CBP60g*, *EDS5* and *PAD3* genes by GTL1. CBP60g binds to and promotes the expression of the SA-biosynthesis gene *ICS1* and the SA-signaling *NPR1* [59]. Furthermore, WRKY33 and MKS1 are two downstream target proteins of flg22-activated MPK4 that mutually regulate the expression of *PAD3* encoding an enzyme required for synthesis of antimicrobial camalexin [61, 64]. Recently, it was shown that the MEKK1-MKK1/2-MPK4 cascade is guarded by the NB-LRR gene SUMM2 and that the guardee of this system is CRCK3 which directly interacts with SUMM2 [32]. The double mutant of *mpk4/summ2* is to some extent suppressed in the *mpk4* autoimmune phenotype and partially restored in growth. Eventually, these data explain the severe *mpk4* mutant phenotypes and suggest that MPK4 acts actually as a positive regulator of defense. However, the only partial suppression indicates that MPK4 is involved in immune and growth regulation independently of SUMM2. The up-regulation of *PAD3* is unaffected in the *summ2* mutant after flg22-application [34]. Unlike the *gtl1-2* mutant, the compromised up-regulation of *PAD3* after flg22-treatment refers directly to the cooperation of MPK4 and GTL1 in a SUMM2 independent manner.

However, MPK4 also negatively regulates defense genes as evidenced by the fact that expression of a constitutively active version of MPK4 results in pathogen hypersensitivity [37]. Moreover, a negative role of MPK4 in defense gene expression is also provided by the work on the transcriptional repressor ASR3, whereby PAMP-induced MPK4 phosphorylation of ASR3 was shown to enhance its DNA binding and repression of a considerable number of defense target genes [36]. ASR3 acts as a transcriptional repressor through its EAR motif and displays opposite *FRK1* regulation as GTL1. Interestingly, ASR3 is also a member of the plant-specific trihelix transcription factor family but belongs to an SH4 clade. The *asr3* mutant shows, unlike *gtl1*, an enhanced resistance against virulent bacterial strains. By contrast, the susceptibility of *asr3* to infection by the avirulent strain *PstDC3000 avrRpt2* matches WT plants. Pathogen resistance to *PstDC3000 AvrRpt2* and *PstDC3000 AvrRpm1* is triggered upon perception by the CC-NB-LRR receptor. The fact that *gtl1* exhibits an enhanced susceptibility to either *PstDC3000* strains demonstrates that both trihelix TF family members do not act redundantly and exert distinct and opposite biological functions in immunity.

Interestingly, GTL1 was recently also shown to alter drought tolerance of *Arabidopsis* [46], and the underlying mechanism was suggested to be due to the altered, reduced number of stomates in *gtl1* plants making them more robust under drought conditions. GTL1 is assumed to monitor the water status in plants to determine the most appropriate number of stomates during plant development. This effect was shown to be exerted through the repression of the *SDD1* gene as a direct target of GTL1.

In summary, the current data suggest that fine-tuning of GTL1 activity plays an important role in defining the balance between growth, defense and developmental adaptations to biotic and abiotic stress conditions. Given its involvement and role in these processes, further studies are warranted into the regulation of GTL1 at the post-translational level.

### GTL1 contribute to RIN4-mediated Effector-triggered immunity

RPM1-INTERACTING PROTEIN 4 (RIN4) interacts with AvrRpm1 and *Pseudomonas syringae pv maculicola 1* (RPM1) [5, 65], whereby the association of AvrRpm1 provokes the phosphorylation of RIN4 by RIN4-interacting receptor-like protein kinase (RIPK) [13] enhancing its activity as a negative regulator of plant defense. However, phosphorylated RIN4 induces the activation of the R-protein RPM1 triggering the RPM1-dependent defense response [13]. MPK4 is a crucial regulator of defense against virulent pathogens and PTI, but the protein kinase is also implicated in ETI regulation [37]. Consistent with a role of *GTL1* in ETI, resistance of *gtl1* plants infected with the avirulent *Pst AvrRpm1* and *Pst AvrRpt2* strain was compromised in the *gtl1* background, indicating that *GTL1* is a positive regulator of RPM1/RPT2-mediated ETI. Interestingly, *mpk4* mutants complemented by the constitutively active MPK4 (CA-MPK4) exhibit distinct responses to different avirulent *Pseudomonas* strains. CA-MPK4 lines are affected in pathogen resistance mediated by TIR-NB-LRR, but not CC-NB-LRR, receptors. In this regard, CA-MPK4 lines retained WT-like resistance to *Pst DC3000 AvrRpm1* recognised by CC-NB-LRR receptors, whereas we showed that the *mpk4* mutant is more resistance, while the *mpk4/gtl1* mutant partially restored susceptibility. Therefore, we postulate that MPK4 functions as a negative regulator of GTL1 in *AvrRpm1* -triggered RIN4-mediated immunity. In summary, we reason that GTL1 is embedded in the MPK4 pathway and coordinates SA-metabolism and homeostasis which directly impacts basal immunity, PAMP- and effector-triggered immunity.

## Materials and methods

### Plant material and growth conditions

Experiments were performed by the usage of *Arabidopsis thaliana* of the Columbia accession grown on soil in plant growth chambers (Percival Scientific) under short-day conditions (8h light/ 16 h dark) at 22°C. *Nicotiana benthamiana* were grown under long-day conditions (16 h light + 8 h darkness) at 28°C. *gtl1-2 (Salk_005965)*, *gtl1-5 (Salk_044308) and mpk4-2 (Salk_056245)* seeds were obtained from NASC.

### Accession numbers

*GTL1* (AT1G33240), *MPK4* (AT4G01370).

### Additional Materials and Methods

See **S1 Materials and Methods**

## Supporting information

S1 Fig Domain map of GTL1 and *in-vitro* kinase assay. **A)** Schematic representation of GTL1; NTH-Myb-like, N-terminal trihelix domain; CC, Coiled coil domain; CTH-Myb-like, C-terminal trihelix domain; red bar, putative MAPK interaction domain.
**B)** In *in-vitro* kinase assays followed by LC/MS-MS, MPK4 does not phosphorylate GTL1 at the previously reported phosphopeptide nor at another site.
(TIF)

S2 Fig Phenotypical and functional characterization of *gtl1* and *GTL1ox* lines. **A)** Overview of the phenotype and shoot-fresh weight of WT, *gtl1-2*, *gtl1-5*, *GTL1ox1 and GTL1ox2* plants. The shoot fresh weight was analyzed of 2 week-old plants in 3 biological replicates. Error bars, mean ± SEM, statistical significance was analyzed by Student’s test; n.s, non-significant against WT. Scale bar = 1cm.
**B)** The allelic *GTL1* mutants *gtl1-2 and gtl1-5* were challenged by leaf infiltration with *PstDC3000*. Plants, of three biological replicates, were leaf-infiltrated with a bacterial suspension at OD_600_ 0.005, the density of colony-forming units (cfu) was analyzed 2 and 72 hours post inoculation (hpi). Error bars, mean ± SEM, statistical significance was analyzed by Student’s test, asterisks indicate significant differences compared to treated WT, * *p* ≤ 0.05, ** *p* ≤ 0.01, *** *p* ≤ 0.001.
**C)** Pathogen-treatment of *GTL1ox1* and *GTL1ox2* lines refers to **Fig 1F**.
**D)** Negative control for ROS-burst assay (**Fig 2E**)
**E)** Expression of *PAD3* and *CAD8* after SA application. 14 day-old WT seedlings were treated with 1μM SA for 6 hours. Error bars, mean ± SEM, Asterisks indicate significant differences compared to untreated WT, * *p* ≤ 0.05,*** *p* ≤ 0.001.
(TIF)

S3 Fig Expression of *CBP60g*, *CAD8*, *PAD3* and *FRK1*. **A-B)** Expression of *CBP60g* **(A)**, *PAD3* **(B)** and *CAD8* **(C)** in *gtl1-2* and *mpk4*.
**D)** Expression of *FRK1* after SA-treatment. 14 day-old WT seedlings were treated with 1μM SA for 6 hours. Error bars, mean ± SEM, statistical significance was analyzed by Student’s test, letters above bars represent significance groups, *p* ≤ 0.01.
(TIF)

S4 Fig Expression of *CBP60g*, *EDS5* and *PAD3* and further pathogen-treatments of *GTL1ox* lines. **A)** GTL1_GFP fusion protein and single GFP in *Arabidopsis* root of independent transgenic lines used for ChIP.
**B-D)** Expression of *CBP60g* (**B**) and *EDS5* (**C**) *and PAD3* (**D**) in *gtl1-2*, *GTL1ox1* and *GTL1ox2* after flg22 application (1μM, flg22 for 1hr). Statistical significance was analyzed by Student’s test. Letters above bars represent significance groups, *p* ≤ 0.05; n.s, non-significant.
**E-F)** Pathogen-treatment of *GTL1ox1* and *GTL1ox2*, refers to **Fig 6A and 6B**.
**G)** WT, *gtl1-2*, *mpk4-2* and *mpk4/gtl1* double mutant. The shoot fresh weight was analyzed of 7 week-old plants in 3 biological replicates. Error bars, mean ± SEM, statistical significance was analyzed by Student’s test. Letters above bars represent significance groups, *p*≤ 0.001.
(TIF)

S1 Table Transcriptome composition of *gtl1-2* /WT of 14 day-old seedlings. GO terms of up- and down-regulated genes. Isolation of GTL1 in the in vivo MPK4-Tandem Affinity Purification approach combined with an LC-MS/MS analysis before and after flg22 application.
(XLSX)

S2 Table Genes dedicated to cluster I and II, GO term analysis, Matrix-cluster. (XLSX)

S3 Table Transcriptome composition of *gtl1-2* /WT of 14 day-old seedlings, 1 hr after flg22-treatment, *p*≤0.0001, Go term analysis of down-regulated genes. (XLSX)

S1 Materials and Methods (DOCX)

## References

[1] Tsuda K. and Katagiri F., Comparing signaling mechanisms engaged in pattern-triggered and effector-triggered immunity. Curr Opin Plant Biol, 2010 13(4): p. 459–65. 10.1016/j.pbi.2010.04.006 20471306

[2] Nakagami H., Pitzschke A., and Hirt H., Emerging MAP kinase pathways in plant stress signalling. Trends Plant Sci, 2005 10(7): p. 339–46. 10.1016/j.tplants.2005.05.009 15953753

[3] Chen H., et al, A Bacterial Type III Effector Targets the Master Regulator of Salicylic Acid Signaling, NPR1, to Subvert Plant Immunity. Cell Host Microbe, 2017 22(6): p. 777–788 e7. 10.1016/j.chom.2017.10.019 29174403

[4] Eschen-Lippold L., et al, Bacterial AvrRpt2-Like Cysteine Proteases Block Activation of the Arabidopsis Mitogen-Activated Protein Kinases, MPK4 and MPK11. Plant Physiol, 2016 171(3): p. 2223–38. 10.1104/pp.16.00336 27208280 PMC4936563

[5] Mackey D., et al, RIN4 interacts with Pseudomonas syringae type III effector molecules and is required for RPM1-mediated resistance in Arabidopsis. Cell, 2002 108(6): p. 743–54. 11955429 10.1016/s0092-8674(02)00661-x

[6] Belkhadir Y., et al, Arabidopsis RIN4 negatively regulates disease resistance mediated by RPS2 and RPM1 downstream or independent of the NDR1 signal modulator and is not required for the virulence functions of bacterial type III effectors AvrRpt2 or AvrRpm1. Plant Cell, 2004 16(10): p. 2822–35. 10.1105/tpc.104.024117 15361584 PMC520974

[7] Wu L., et al, Go in for the kill: How plants deploy effector-triggered immunity to combat pathogens. *[Corrected]* Virulence, 2014 5(7): p. 710–21. 10.4161/viru.29755 25513772 PMC4189877

[8] Bisgrove S.R., et al, A disease resistance gene in Arabidopsis with specificity for two different pathogen avirulence genes. Plant Cell, 1994 6(7): p. 927–33. 10.1105/tpc.6.7.927 8069104 PMC160489

[9] Grant M.R., et al, Structure of the Arabidopsis RPM1 gene enabling dual specificity disease resistance. Science, 1995 269(5225): p. 843–6. 7638602 10.1126/science.7638602

[10] Boyes D.C., Nam J., and Dangl J.L., The Arabidopsis thaliana RPM1 disease resistance gene product is a peripheral plasma membrane protein that is degraded coincident with the hypersensitive response. Proc Natl Acad Sci U S A, 1998 95(26): p. 15849–54. 9861059 10.1073/pnas.95.26.15849 PMC28133

[11] Kim M.G., et al, Two Pseudomonas syringae type III effectors inhibit RIN4-regulated basal defense in Arabidopsis. Cell, 2005 121(5): p. 749–59. 10.1016/j.cell.2005.03.025 15935761

[12] Deslandes L. and Rivas S., Catch me if you can: bacterial effectors and plant targets. Trends Plant Sci, 2012 17(11): p. 644–55. 10.1016/j.tplants.2012.06.011 22796464

[13] Liu J., et al, A receptor-like cytoplasmic kinase phosphorylates the host target RIN4, leading to the activation of a plant innate immune receptor. Cell Host Microbe, 2011 9(2): p. 137–46. 10.1016/j.chom.2011.01.010 21320696 PMC3070605

[14] Chung E.H., et al, Specific threonine phosphorylation of a host target by two unrelated type III effectors activates a host innate immune receptor in plants. Cell Host Microbe, 2011 9(2): p. 125–36. 10.1016/j.chom.2011.01.009 21320695 PMC3061827

[15] Mackey D., et al, Arabidopsis RIN4 is a target of the type III virulence effector AvrRpt2 and modulates RPS2-mediated resistance. Cell, 2003 112(3): p. 379–89. 12581527 10.1016/s0092-8674(03)00040-0

[16] Kim H.S., et al, The Pseudomonas syringae effector AvrRpt2 cleaves its C-terminally acylated target, RIN4, from Arabidopsis membranes to block RPM1 activation. Proc Natl Acad Sci U S A, 2005 102(18): p. 6496–501. 10.1073/pnas.0500792102 15845764 PMC1088372

[17] Dempsey D.A. and Klessig D.F., Salicylic acid, active oxygen species and systemic acquired resistance in plants. Trends Cell Biol, 1994 4(9): p. 334–8. 14731471 10.1016/0962-8924(94)90235-6

[18] Durrant W.E. and Dong X., Systemic acquired resistance. Annu Rev Phytopathol, 2004 **42**: p. 185–209. 10.1146/annurev.phyto.42.040803.140421 15283665

[19] Bigeard J., Colcombet J., and Hirt H., Signaling mechanisms in pattern-triggered immunity (PTI). Mol Plant, 2015 8(4): p. 521–39. 10.1016/j.molp.2014.12.022 25744358

[20] Meng X. and Zhang S., MAPK cascades in plant disease resistance signaling. Annu Rev Phytopathol, 2013 51: p. 245–66. 10.1146/annurev-phyto-082712-102314 23663002

[21] Nitta Y., Ding P., and Zhang Y., Identification of additional MAP kinases activated upon PAMP treatment. Plant Signal Behav, 2014 9(11): p. e976155 10.4161/15592324.2014.976155 25482788 PMC4623049

[22] Frei dit Frey N., et al, Functional analysis of Arabidopsis immune-related MAPKs uncovers a role for MPK3 as negative regulator of inducible defences. Genome Biol, 2014 15(6): p. R87 10.1186/gb-2014-15-6-r87 24980080 PMC4197828

[23] Asai T., et al, MAP kinase signalling cascade in Arabidopsis innate immunity. Nature, 2002 415(6875): p. 977–83. 10.1038/415977a 11875555

[24] Ichimura K., et al, MEKK1 is required for MPK4 activation and regulates tissue-specific and temperature-dependent cell death in Arabidopsis. J Biol Chem, 2006 281(48): p. 36969–76. 10.1074/jbc.M605319200 17023433

[25] Nakagami H., et al, A Mitogen-activated protein kinase kinase kinase mediates reactive oxygen species homeostasis in Arabidopsis. J Biol Chem, 2006 281(50): p. 38697–704. 10.1074/jbc.M605293200 17043356

[26] Gao M., et al, MEKK1, MKK1/MKK2 and MPK4 function together in a mitogen-activated protein kinase cascade to regulate innate immunity in plants. Cell Res, 2008 18(12): p. 1190–8. 10.1038/cr.2008.300 18982020

[27] Qiu J.L., et al, Arabidopsis mitogen-activated protein kinase kinases MKK1 and MKK2 have overlapping functions in defense signaling mediated by MEKK1, MPK4, and MKS1. Plant Physiol, 2008 148(1): p. 212–22. 10.1104/pp.108.120006 18599650 PMC2528087

[28] Liu Y. and Zhang S., Phosphorylation of 1-aminocyclopropane-1-carboxylic acid synthase by MPK6, a stress-responsive mitogen-activated protein kinase, induces ethylene biosynthesis in Arabidopsis. Plant Cell, 2004 16(12): p. 3386–99. 10.1105/tpc.104.026609 15539472 PMC535880

[29] Ren D., et al, A fungal-responsive MAPK cascade regulates phytoalexin biosynthesis in Arabidopsis. Proc Natl Acad Sci U S A, 2008 105(14): p. 5638–43. 10.1073/pnas.0711301105 18378893 PMC2291085

[30] Xu J., et al, Pathogen-Responsive MPK3 and MPK6 Reprogram the Biosynthesis of Indole Glucosinolates and Their Derivatives in Arabidopsis Immunity. Plant Cell, 2016 28(5): p. 1144–62. 10.1105/tpc.15.00871 27081184 PMC4904669

[31] Petersen M., et al, Arabidopsis map kinase 4 negatively regulates systemic acquired resistance. Cell, 2000 103(7): p. 1111–20. 11163186 10.1016/s0092-8674(00)00213-0

[32] Zhang Z., et al, The NLR protein SUMM2 senses the disruption of an immune signaling MAP kinase cascade via CRCK3. EMBO Rep, 2017 18(2): p. 292–302. doi: 10.15252/embr.201642704 27986791 10.15252/embr.201642704 PMC5286374

[33] Roux M.E., et al, The mRNA decay factor PAT1 functions in a pathway including MAP kinase 4 and immune receptor SUMM2. EMBO J, 2015 34(5): p. 593–608. doi: 10.15252/embj.201488645 25603932 10.15252/embj.201488645 PMC4365030

[34] Zhang Z., et al, Disruption of PAMP-induced MAP kinase cascade by a Pseudomonas syringae effector activates plant immunity mediated by the NB-LRR protein SUMM2. Cell Host Microbe, 2012 11(3): p. 253–63. 10.1016/j.chom.2012.01.015 22423965

[35] Kong Q., et al, The MEKK1-MKK1/MKK2-MPK4 kinase cascade negatively regulates immunity mediated by a mitogen-activated protein kinase kinase kinase in Arabidopsis. Plant Cell, 2012 24(5): p. 2225–36. 10.1105/tpc.112.097253 22643122 PMC3442598

[36] Li B., et al, Phosphorylation of trihelix transcriptional repressor ASR3 by MAP KINASE4 negatively regulates Arabidopsis immunity. Plant Cell, 2015 27(3): p. 839–56. 10.1105/tpc.114.134809 25770109 PMC4558661

[37] Berriri S., et al, Constitutively active mitogen-activated protein kinase versions reveal functions of Arabidopsis MPK4 in pathogen defense signaling. Plant Cell, 2012 24(10): p. 4281–93. 10.1105/tpc.112.101253 23115249 PMC3517250

[38] Smalle J., et al, The trihelix DNA-binding motif in higher plants is not restricted to the transcription factors GT-1 and GT-2. Proc Natl Acad Sci U S A, 1998 95(6): p. 3318–22. 9501260 10.1073/pnas.95.6.3318 PMC19739

[39] Kaplan-Levy R.N., et al, The trihelix family of transcription factors—light, stress and development. Trends Plant Sci, 2012 17(3): p. 163–71. 10.1016/j.tplants.2011.12.002 22236699

[40] Nagano Y., et al, Trihelix DNA-binding protein with specificities for two distinct cis-elements: both important for light down-regulated and dark-inducible gene expression in higher plants. J Biol Chem, 2001 276(25): p. 22238–43. 10.1074/jbc.M102474200 11301338

[41] Dehesh K., et al, GT-2: a transcription factor with twin autonomous DNA-binding domains of closely related but different target sequence specificity. EMBO J, 1992 11(11): p. 4131–44. 1396594 10.1002/j.1460-2075.1992.tb05506.x PMC556923

[42] Zhou D.X., Regulatory mechanism of plant gene transcription by GT-elements and GT-factors. Trends Plant Sci, 1999 4(6): p. 210–214. 10366876 10.1016/s1360-1385(99)01418-1

[43] Ni M., et al, GT-2: in vivo transcriptional activation activity and definition of novel twin DNA binding domains with reciprocal target sequence selectivity. Plant Cell, 1996 8(6): p. 1041–59. 10.1105/tpc.8.6.1041 8672890 PMC161160

[44] Breuer C., et al, The trihelix transcription factor GTL1 regulates ploidy-dependent cell growth in the Arabidopsis trichome. Plant Cell, 2009 21(8): p. 2307–22. 10.1105/tpc.109.068387 19717615 PMC2751941

[45] Breuer C., et al, Transcriptional repression of the APC/C activator CCS52A1 promotes active termination of cell growth. EMBO J, 2012 31(24): p. 4488–501. 10.1038/emboj.2012.294 23143274 PMC3545286

[46] Yoo C.Y., et al, The Arabidopsis GTL1 transcription factor regulates water use efficiency and drought tolerance by modulating stomatal density via transrepression of SDD1. Plant Cell, 2010 22(12): p. 4128–41. 10.1105/tpc.110.078691 21169508 PMC3027182

[47] Dinkel H., et al, ELM 2016—data update and new functionality of the eukaryotic linear motif resource. Nucleic Acids Res, 2016 44(D1): p. D294–300. 10.1093/nar/gkv1291 26615199 PMC4702912

[48] Reiland S., et al, Large-scale Arabidopsis phosphoproteome profiling reveals novel chloroplast kinase substrates and phosphorylation networks. Plant Physiol, 2009 150(2): p. 889–903. 10.1104/pp.109.138677 19376835 PMC2689975

[49] Reiland S., et al, Comparative phosphoproteome profiling reveals a function of the STN8 kinase in fine-tuning of cyclic electron flow (CEF). Proc Natl Acad Sci U S A, 2011 108(31): p. 12955–60. 10.1073/pnas.1104734108 21768351 PMC3150903

[50] Wang P., et al, Quantitative phosphoproteomics identifies SnRK2 protein kinase substrates and reveals the effectors of abscisic acid action. Proc Natl Acad Sci U S A, 2013 110(27): p. 11205–10. 10.1073/pnas.1308974110 23776212 PMC3703982

[51] Choudhary M.K., et al, Quantitative Circadian Phosphoproteomic Analysis of Arabidopsis Reveals Extensive Clock Control of Key Components in Physiological, Metabolic, and Signaling Pathways. Mol Cell Proteomics, 2015 14(8): p. 2243–60. 10.1074/mcp.M114.047183 26091701 PMC4528250

[52] Roitinger E., et al, Quantitative phosphoproteomics of the ataxia telangiectasia-mutated (ATM) and ataxia telangiectasia-mutated and rad3-related (ATR) dependent DNA damage response in Arabidopsis thaliana. Mol Cell Proteomics, 2015 14(3): p. 556–71. 10.1074/mcp.M114.040352 25561503 PMC4349977

[53] Bigeard J., et al, Proteomic and phosphoproteomic analyses of chromatin-associated proteins from Arabidopsis thaliana. Proteomics, 2014 14(19): p. 2141–55. 10.1002/pmic.201400072 24889360

[54] Rayapuram N., et al, Quantitative phosphoproteomic analysis reveals shared and specific targets of Arabidopsis MPK3, MPK4 and MPK6. Mol Cell Proteomics, 2017. 10.1074/mcp.RA117.000135 PMC5750851 29167316

[55] Carol R.J., et al, A RhoGDP dissociation inhibitor spatially regulates growth in root hair cells. Nature, 2005 438(7070): p. 1013–6. 10.1038/nature04198 16355224

[56] Shigeto J. and Tsutsumi Y., Diverse functions and reactions of class III peroxidases. New Phytol, 2016 209(4): p. 1395–402. 10.1111/nph.13738 26542837

[57] Zhang R., et al, A high quality Arabidopsis transcriptome for accurate transcript-level analysis of alternative splicing. Nucleic Acids Res, 2017 45(9): p. 5061–5073. 10.1093/nar/gkx267 28402429 PMC5435985

[58] Wang L., et al, CBP60g and SARD1 play partially redundant critical roles in salicylic acid signaling. Plant J, 2011 67(6): p. 1029–41. 10.1111/j.1365-313X.2011.04655.x 21615571

[59] Sun T., et al, ChIP-seq reveals broad roles of SARD1 and CBP60g in regulating plant immunity. Nat Commun, 2015 6: p. 10159 10.1038/ncomms10159 27206545 PMC4703862

[60] Zhou N., Tootle T.L., and Glazebrook J., Arabidopsis PAD3, a gene required for camalexin biosynthesis, encodes a putative cytochrome P450 monooxygenase. Plant Cell, 1999 11(12): p. 2419–28. 10590168 10.1105/tpc.11.12.2419 PMC144139

[61] Schuhegger R., et al, CYP71B15 (PAD3) catalyzes the final step in camalexin biosynthesis. Plant Physiol, 2006 141(4): p. 1248–54. 10.1104/pp.106.082024 16766671 PMC1533948

[62] Somssich I.E., et al, Arabidopsis thaliana defense-related protein ELI3 is an aromatic alcohol:NADP+ oxidoreductase. Proc Natl Acad Sci U S A, 1996 93(24): p. 14199–203. 11038530 10.1073/pnas.93.24.14199 PMC19517

[63] Nawrath C., et al, EDS5, an essential component of salicylic acid-dependent signaling for disease resistance in Arabidopsis, is a member of the MATE transporter family. Plant Cell, 2002 14(1): p. 275–86. 10.1105/tpc.010376 11826312 PMC150564

[64] Qiu J.L., et al, Arabidopsis MAP kinase 4 regulates gene expression through transcription factor release in the nucleus. EMBO J, 2008 27(16): p. 2214–21. 10.1038/emboj.2008.147 18650934 PMC2519101

[65] El Kasmi F., et al, Signaling from the plasma-membrane localized plant immune receptor RPM1 requires self-association of the full-length protein. Proc Natl Acad Sci U S A, 2017 114(35): p. E7385–E7394. 10.1073/pnas.1708288114 28808003 PMC5584451

