# Supplementary figures and images for: The Trihelix transcription factor GT2-like 1 (GTL1) promotes salicylic acid metabolism, and regulates bacterial-triggered immunity
Source: PLoS Genet. 2018 Oct 23;14(10):e1007708. doi: 10.1371/journal.pgen.1007708 (PMC6198943; doi:10.1371/journal.pgen.1007708)

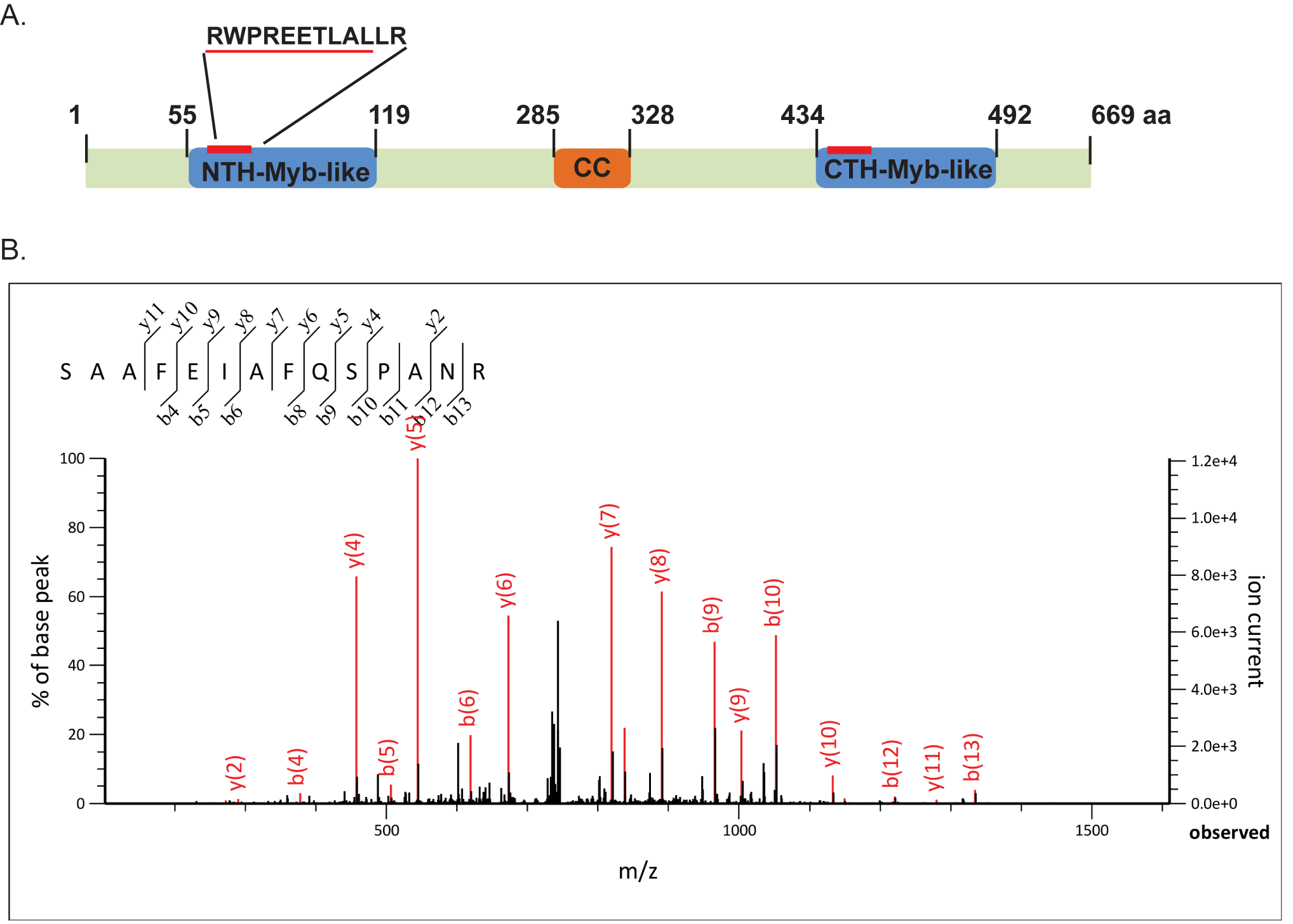

Supplement: S1 Fig — A) Schematic representation of GTL1; NTH-Myb-like, N-terminal trihelix domain; CC, Coiled coil domain; CTH-Myb-like, C-terminal trihelix domain; red bar, putative MAPK interaction domain. B) In in-vitro kinase assays followed by LC/MS-MS, MPK4 does not phosphorylate GTL1 at the previously reported phosphopeptide nor at another site. (TIF) [file pgen.1007708.s001.tif]

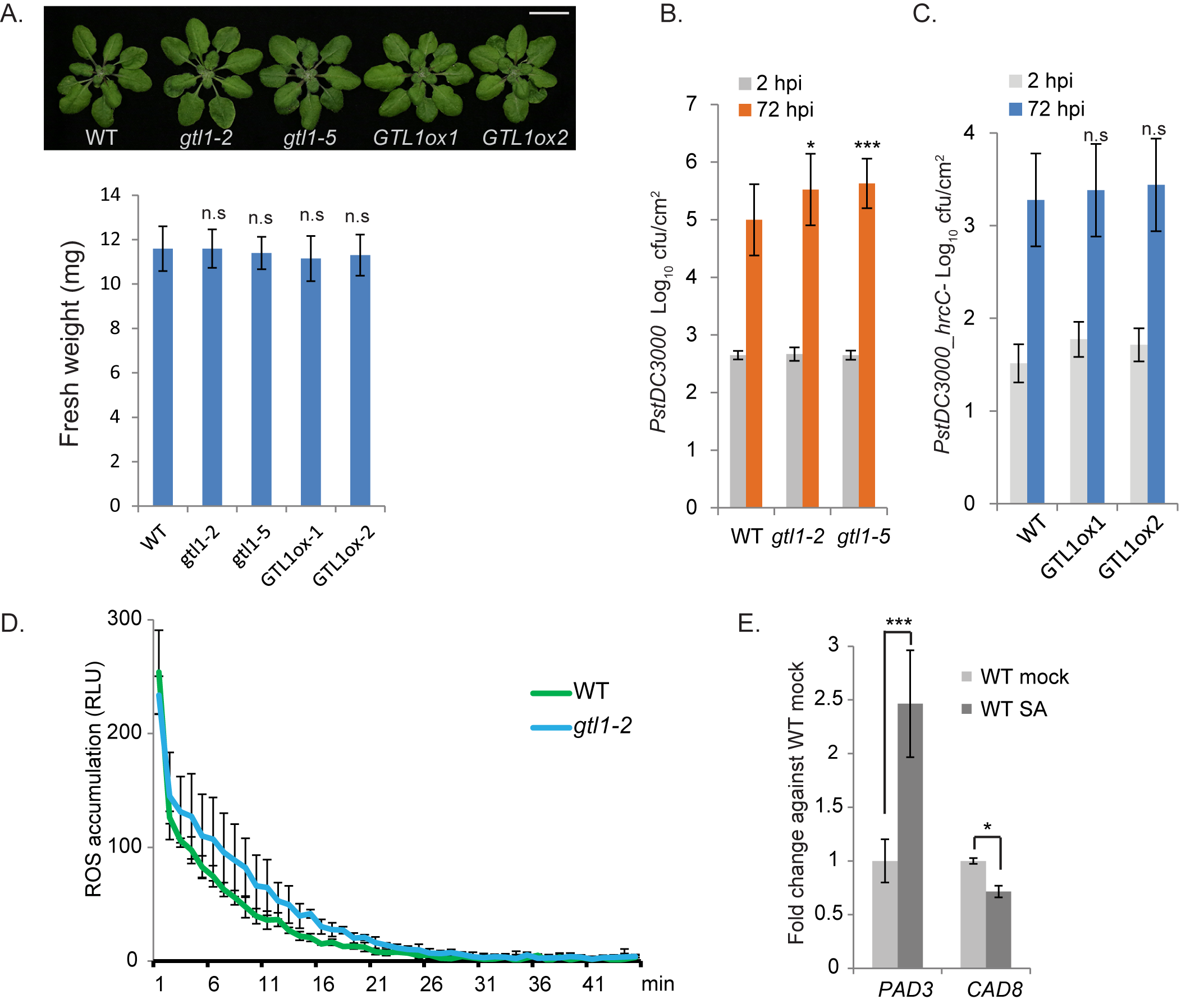

Supplement: S2 Fig — A) Overview of the phenotype and shoot-fresh weight of WT, gtl1-2, gtl1-5, GTL1ox1 and GTL1ox2 plants. The shoot fresh weight was analyzed of 2 week-old plants in 3 biological replicates. Error bars, mean ± SEM, statistical significance was analyzed by Student’s test; n.s, non-significant against WT. Scale bar = 1cm. B) The allelic GTL1 mutants gtl1-2 and gtl1-5 were challenged by leaf infiltration with PstDC3000. Plants, of three biological replicates, were leaf-infiltrated with a bacterial suspension at OD600 0.005, the density of colony-forming units (cfu) was analyzed 2 and 72 hours post inoculation (hpi). Error bars, mean ± SEM, statistical significance was analyzed by Student’s test, asterisks indicate significant differences compared to treated WT, * p ≤ 0.05, ** p ≤ 0.01, *** p ≤ 0.001. C) Pathogen-treatment of GTL1ox1 and GTL1ox2 lines refers to Fig 1F. D) Negative control for ROS-burst assay (Fig 2E) E) Expression of PAD3 and CAD8 after SA application. 14 day-old WT seedlings were treated with 1μM SA for 6 hours. Error bars, mean ± SEM, Asterisks indicate significant differences compared to untreated WT, * p ≤ 0.05,*** p ≤ 0.001. (TIF) [file pgen.1007708.s002.tif]

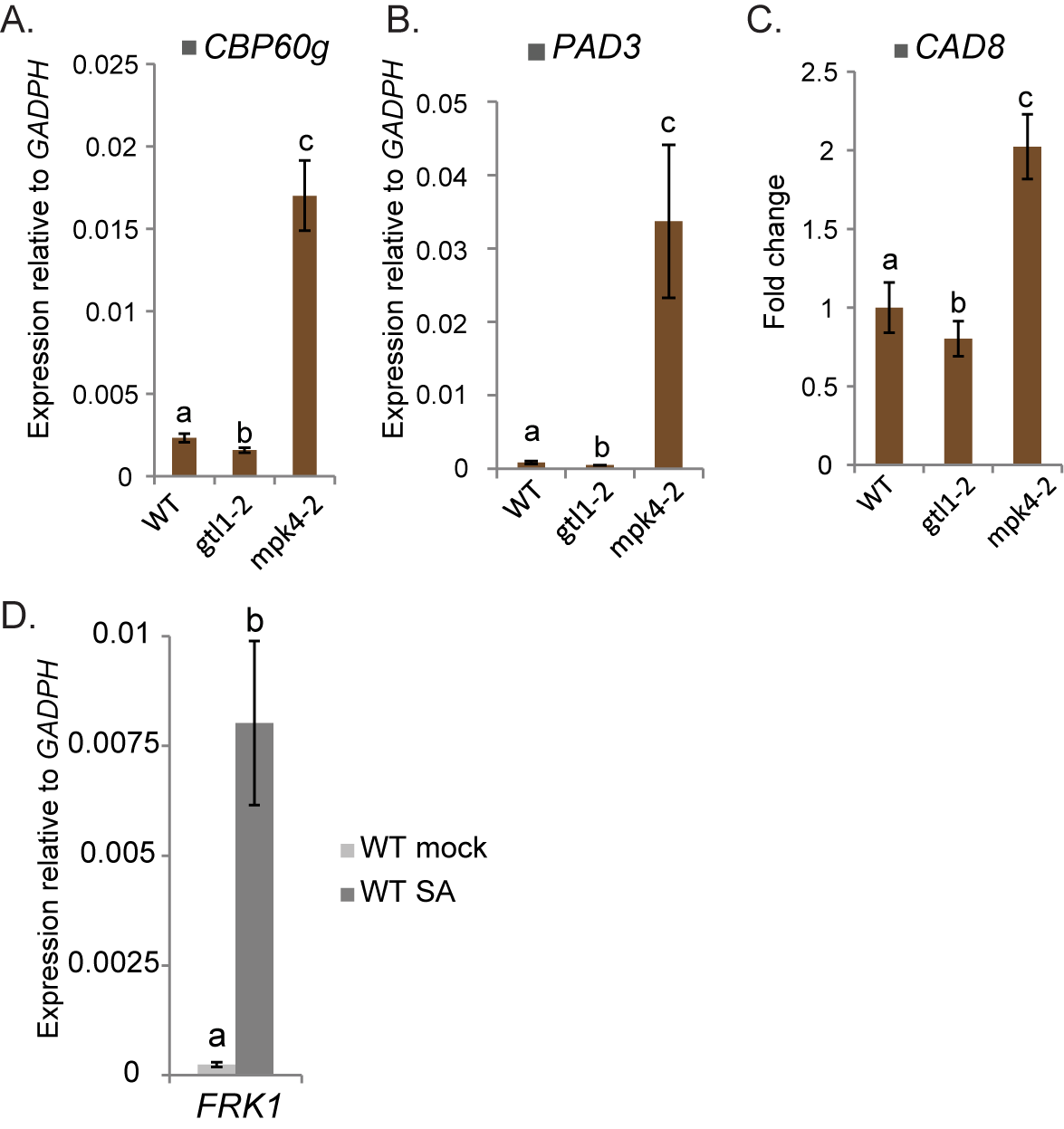

Supplement: S3 Fig — A-B) Expression of CBP60g (A), PAD3 (B) and CAD8 (C) in gtl1-2 and mpk4. D) Expression of FRK1 after SA-treatment. 14 day-old WT seedlings were treated with 1μM SA for 6 hours. Error bars, mean ± SEM, statistical significance was analyzed by Student’s test, letters above bars represent significance groups, p ≤ 0.01. (TIF) [file pgen.1007708.s003.tif]

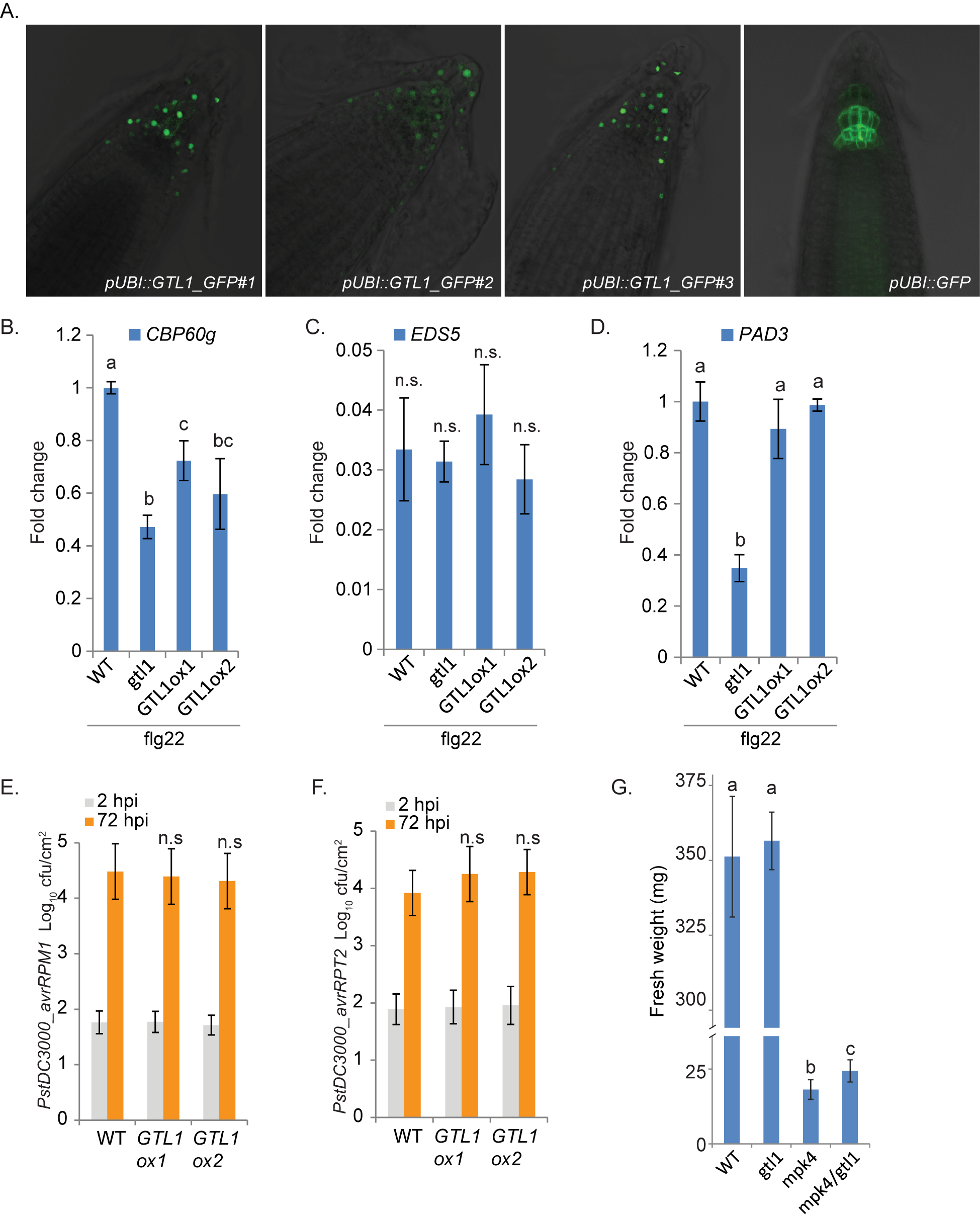

Supplement: S4 Fig — A) GTL1_GFP fusion protein and single GFP in Arabidopsis root of independent transgenic lines used for ChIP. B-D) Expression of CBP60g (B) and EDS5 (C) and PAD3 (D) in gtl1-2, GTL1ox1 and GTL1ox2 after flg22 application (1μM, flg22 for 1hr). Statistical significance was analyzed by Student’s test. Letters above bars represent significance groups, p ≤ 0.05; n.s, non-significant. E-F) Pathogen-treatment of GTL1ox1 and GTL1ox2, refers to Fig 6A and 6B. G) WT, gtl1-2, mpk4-2 and mpk4/gtl1 double mutant. The shoot fresh weight was analyzed of 7 week-old plants in 3 biological replicates. Error bars, mean ± SEM, statistical significance was analyzed by Student’s test. Letters above bars represent significance groups, p≤ 0.001. (TIF) [file pgen.1007708.s004.tif]
