# Supplementary material for: The Trihelix transcription factor GT2-like 1 (GTL1) promotes salicylic acid metabolism, and regulates bacterial-triggered immunity
Source: PLoS Genet. 2018 Oct 23;14(10):e1007708. doi: 10.1371/journal.pgen.1007708 (PMC6198943; doi:10.1371/journal.pgen.1007708)
Supplement: S1 Materials and Methods — (DOCX) [file pgen.1007708.s008.docx]

**Supplemental Materials and Methods:**

**RNA Extraction and real-time quantitative PCR analysis**

Total RNAs were extracted from 14 day-old seedlings, grown on half-strength Murashige and Skoog (MS) plates, with the NucleoSpin® RNA Plant (MACHEREY-NAGEL), according to the manufacturer's instructions. First strand cDNA was synthesised from 5μg of total RNAs by the use of SuperScript® First-Strand Synthesis System for RT-PCR (Lifetechnology) according to the manufacturer’s instructions. The cDNA stock was diluted to a final concentration of 25ng/µl. Subsequently, 500nM of each primer was applied and mixed with LightCycler® 480 Sybr Green I Master mix (Roche Applied Science) for quantitative PCR analysis, according to the manufacturer's instructions. Products were amplified and fluorescent signals acquired with a LightCycler® 480 detection system. The specificity of amplification products was determined by melting curves. GADPH was used as internal control for signal normalisation. Exor4 relative quantification software (Roche Applied Science) automatically calculated the relative expression level of the selected genes with algorithms based on the ΔΔCt method. Data were used from duplicates of at least three biological replicates.

**The primer used in this study**

| 392s | GGCGAGAAGTGAAGCTTTTG | *qPCR CBP60g* |
| --- | --- | --- |
| 392as | GCACGGAGGATGATGTTTTT | *qPCR CBP60g* |
| 416s | GAAGCCACTCGAGCTACCTG | *qPCR CAD8* |
| 416as | GTGTTTCCCGGCCATATCTA | *qPCR CAD8* |
| 175s | ACGGGCATAGTTCCACAAAG | *qPCR FRK1* |
| 175as | CGTCAAAAGAACGACGATGA | *qPCR FRK1* |
| ksk-145 | TATGGGGGATAAGGGGTTCT | *qPCR ICS1* |
| ksk-146 | GCCCTAGTTACAACCCGAAA | *qPCR ICS1* |
| 140s | GGTCTATATCTACGACGGCTTCTTTC | *qPCR EDS5* |
| 140as | GGACTCGGCCCATCTGAAT | *qPCR EDS5* |
| 87s | GAATCGGTCGTTTGGTTGCTA | *qPCR GADPH* |
| 87as | TTAACAGCGACGAGCTCAACAT | *qPCR GADPH* |
| 427s | tgacacaacacaacacaacaca | *ChIPqPCR CBP60g* |
| 427as | ttccaggaagattcgtgctc | *ChIPqPCR CBP60g* |
| 428s | tttaaaccctggtatgttttgc | *ChIPqPCR CBP60g* |
| 428as | ttttggtaatgaagatttggtga | *ChIPqPCR CBP60g* |
| 429s | ttttccggtttattttcggata | *ChIPqPCR CBP60g* |
| 429as | TCACTTTCTTCACAACTctgtgg | *ChIPqPCR CBP60g* |
| 433s | tttgagaagtgctcagttttgg | *ChIPqPCR PAD3* |
| 433as | tccgctgtagcttgtgaataa | *ChIPqPCR PAD3* |
| 434s | aaagaatttccctctaaaggaatga | *ChIPqPCR PAD3* |
| 434as | ccttgccctgttcttgtgtt | *ChIPqPCR PAD3* |
| 435s | TCCAAAACTCTGGGAAAACG | *ChIPqPCR, qPCR PAD3* |
| 435as | TCAACGGAACTGTCGAGAAA | *ChIPqPCR, qPCR PAD3* |
| 452s | cttgcgttatggttgggttt | *ChIP_qPCR_pEDS5* |
| 452as | ccagtgtgatggcgaaaata | *ChIP_qPCR_pEDS5* |
| 454s | ccctagcgacaaatgacagc | *ChIP_qPCR_pEDS5* |
| 454as | cctgcataattatttagttggtgttc | *ChIP_qPCR_pEDS5* |
| 455s | ttggtctgaagaaaaggcaaa | *ChIP_qPCR_gEDS5* |
| 455as | tcaaatgcttgaaaacggtct | *ChIP_qPCR_gEDS5* |
| mpk4-LP | GTGACAATGCAAGAAGATACGTTAGACAGC | *genotyping mpk4* |
| mpk4-RP | CTTGAAATATCTACAGAGTTGGTGTG | *genotyping mpk4* |
| LBb1 | ATTTTGCCGATTTCGGAAC | *Salk-T-DNA, genotyping mpk4* |
| 29as | TGGTGGTAATGAAGTTGTGGAGG | *genotyping gtl1* |
| 160as | AAGTGGAAGAGGTGGTGGAG | *genotyping gtl1* |
| LBb3 | ATTTTGCCGATTTCGGAAC | *Salk-T-DNA, genotyping gtl1* |

**ChIP Analyses**

Nuclear proteins were extracted from 14 day-old seedlings on half MS medium. After quantification with the Bradford method, equal amounts of proteins were resolved by SDS-PAGE and then transferred to a polyvinylidene difluoride membrane (Bio-Rad) by the use of a Mini-Protean 3 Cell (Bio-Rad). Immunoblot analysis was performed by the use of 1 µg/mL primary polyclonal antibodies raised against GFP (Abcam AB290) and then with secondary antibodies conjugated to alkaline phosphatase. Antibody complexes were detected by chemiluminescence by the use of the Immun-Start AP Substrate kit (Bio-Rad). ChIP assays were performed by the usage of Anti-GFP antibody - ChIP Grade (Abcam) and RNA polymerase II (Santa Cruz) antibodies. Briefly, after plant material fixation in 1% (v/v) formaldehyde, the tissues were homogenised, and the nuclei were isolated and lysed. Cross-linked chromatin was sonicated by the use of a water bath Bioruptor UCD-200 (Diagenode; 15-s on/15-s off pulses, 15 times). The complexes were immunoprecipitated with antibodies overnight at 4°C with gentle shaking and incubated for 1 h at 4°C with 50 μL of Dynabeads Protein A (Invitrogen). Immunoprecipitated DNA was then recovered by the use of the IPure kit (Diagenode) and analysed by qRT-PCR. An aliquot of untreated sonicated chromatin was processed in parallel and used as the total input DNA control.

***In vitro* pulldown/ Immunoblotting**

For in vitro glutathione S-transferase (GST) pull-down assays, *GST*-tagged *MPK3, 4, 6* in the pGEX-6-P vector for bacterial expression and *MBP:His*-tagged *GTL1* in the *pDEST* plasmid were used. For immunoblotting Phospho-p44/42 MAPK (Erk1/2) (Thr202/Tyr204) XP® Rabbit (pTpY) (Cell Signaling) were utilized. The detailed procedure was previously reported [1].

**Bioinformatics analysis of *RNA*seq data**

Sequencing was performed on each library to generate 101-bp paired-end reads on Illumina HiSeq4000 Genome Analyzer platform. Read quality was checked by the use of FastQC and low quality reads were trimmed by the use of the Trimmomatic version 0.32 (http://www.usadellab.org/cms/?page=trimmomatic) with the following parameters: Minimum length of 36 bp; Mean Phred quality score higher than 30; Leading and trailing bases removal with base quality below 3; Sliding window of 4:15. After pre-processing the Illumina reads, the transcript structures were reconstructed by the use of a series of programs, namely, TopHat (ver. 2.1.1; http://tophat.cbcb.umd.edu/) for aligning with the genome, and Cufflinks (ver. 2.2.1; http://cufflinks.cbcb.umd.edu/) for gene structure predictions. For TopHat, the Reference-*Arabidopsis thaliana* (TAIR10) genome (https://www.arabidopsis.org) was used as the reference sequences with a maximum number of mismatches as 2. To identify the differentially expressed genes, the following parameters were used: p-value of 0.05 with a statistical correction by the use of Benjamini Hochberg FDR of 0.05 in cuffdiff. After processing the data, visualisation of differential expression was done by the use of cummeRbund v2.14.0 (http://bioconductor.org/packages/release/bioc/html/cummeRbund.html). Differentially regulated genes that are common among the samples were identified by the use of Venny.

**GO term analysis**

For the GO term analysis, AGRIGO analysis tool was used (<http://bioinfo.cau.edu.cn/agriGO/>, [2] by the use of significantly differentially expressed genes between the tested conditions.

**Plasmid construction**

The coding sequence (cds) of GTL1 in *pDONR207* was synthesized by Genescript®. Whole seedling Arabidopsis cDNA library was used to amplify the cds of the *EXO70B1*. Subsequently, the entry clone was generated by the introduction of the cds in the *pENTR*-vector by the use of the pENTR^TM^ Directional TOPO® Cloning Kit by INVITROGEN according to manufactory’s instruction. Subsequently, entry clones were used to generate protein expression constructs (pDESTMBP [3]) and protein localisation vectors, fused to GFP driven by the Ubiquitin promoter [4].

**Quantification of SA**

Plant materials were lyophilised and ground in a bead beater (Biospec Products, Bartlesville, Okla., USA). Aliquots (about 5 mg dry weight) of powdered tissues were extracted with 400 μL of 10% methanol containing 1% acetic acid and internal standards (11.1 ng of 2H_4_-SA purchased from OlchemIm Ltd., Olomouc, Czech Republic). The samples were extracted in the bead beater for 1 min, placed in ice for 30 min, and then centrifuged at 13,000 g for 10 min at 4^o^C (Eppendorf centrifuge 5424, Hamburg, Germany). The supernatant was carefully removed and the pellet re-extracted with 400 μL of 10% methanol containing 1% acetic acid. Following a further 30 min incubation in ice, the extracts were centrifuged and the supernatants combined. The samples were filtered through 0.22 µm PTFE filters before LC-MS/MS analysis. Analysis SA was performed by the comparison of the retention times and mass transitions with the standards by the use of an Agilent 1200 HPLC (Agilent Technologies, Waldbronn, Germany) coupled to a Q-TRAP 5500 MS (AB SCIEX, MA, USA) with an electrospray source. Chromatographic separation was carried out at 35 ^o^C on a Phenomenex (Torrance, CA, USA) Gemini C18 (150×2.0 mm, 5 μm) column with the solvent system formic acid/acetonitrile/water (0.1/94.9/5, v/v/v; mobile phase A) and formic acid/ acetonitrile/water (0.1/5/94.9, v/v/v; mobile phase B). The gradient used was 0-20 min, 0%-100% A; 20-25 min, 100%A; 25-26 min, 100%-0% A; 26-36 min, 0% A. To reduce contamination of the MS, the first 5 min of the run was directed to waste by the use of the inbuilt Valco valve. Analysis of SA was based on appropriate Multiple Reaction Monitoring (MRM) of ion pairs for labelled and endogenous SA by the use of the following mass transitions: 2H_4_SA 141>97, SA 137>93. The MS was operated in negative ionization mode. The conditions were as follows: Temperature 500 ^o^C, Ion source gas 1 50 psi, Ion source gas 2 60 psi, Ion Spray Voltage -4500 V, curtain gas 40 psi, Collision Gas Medium; DP (-25 V), EP (-9) and CXP (-2) were the same for all compounds. CE (-38), and DT (50) were used for 2H_4_SA and SA. Data were acquired and analysed by the use of Analyst 1.4 software (Applied Biosystems).

***Pseudomonas syringae* Infections**

Plants were spray-inoculated with *Pseudomonas syringae (Pst) DC3000*, *Pst DC3000 ΔavrPto/avrPtoB*, *Pst DC3000 AvrRpm1*, *Pst DC3000 AvrRpt2* and *Pst DC3000 hrcC-* at OD_600_ = 0.2 and sampled 2 h and 72 h after inoculation to determine the level of colonisation as described previously [5] by the analysis of the density of colony-forming units (cfu). In three biological replicates, a total of 30 plants were sampled for each plant genotype by each taking 3 leaf discs per plant. Leaf-infiltrations were performed at a bacterial density of OD_600_ = 0.0005.

**Quantitation of Immunoblot Membranes**

Bradford assays were used to quantify protein levels in extracts and ensure equal loading of total proteins for gels used for immunoblot analysis.

**Kinase assays and phospho-site identification**

Purified recombinant GTL1 protein fused to MBP-tag and constitutively active MPK4 were mixed together in kinase reaction buffer (20 mM Tris-HCl pH 7.5, 10 mM MgCl_2_, 5 mM EGTA, 1 mM DTT and 50 µM ATP) and incubated at ambient temperature for 30 min. SDS-sample buffer was added to stop the reaction followed by the boiling at 95^о^C for 10 min. Protein samples were resolved by SDS-PAGE. The gel was stained with SimplyBlue™ SafeStain (Novex cat. No. LC6065) and the band corresponding to the protein of interest was excised out, cut into small pieces of 0.5 mm3 and destained with four successive washes of 15 min each with ACN and 100 mM NH_4_HCO_3_. Proteins were reduced with 10 mM Tris(2-carboxyethyl)phosphine (TCEP, C-4706 Sigma) in 100 mM NH_4_HCO_3_ at 37^о^C for 1 h followed by alkylation with 20 mM S-Methyl methanethiosulfonate (MMTS, 64306 Sigma) at ambient temperature for 30 min. Proteins were then digested with trypsin (Porcine trypsin, Promega) at 37^о^C overnight. The digestion was stopped by the addition of 1% formic acid, and the peptides were recovered by the incubation of the gel pieces in ACN. The recovered peptide solution was desalted by the use of C18 ZipTip® (Millipore Cat. No. ZTC18S096) and analysed by LC-MS/MS. Briefly, peptide samples were separated on a C18 column (Acclaim PepMap C18, 25 cm length x 75 µm I.D. x 3 µm particle size, 100 Å porosity, Dionex) connected to an LTQ-Orbitrap Velos or a Q-Exactive HF instrument. The LC gradient ramped from 5% solvent B (water/ACN/formic acid, 20/80/0.1, v/v/v) to 45% solvent B over 45 min, then to 90% solvent B for 10 min. The MS instrument acquired fragmentation spectra on the top 10 peptides by the use of CID fragmentation in the LTQ-Orbitrap or HCD in the Q-Exactive instrument. RAW data files obtained were converted to MGF files by the use of Proteome Discoverer interface (version 1.4). Database searches were performed with the Mascot server v2.4 specifying the following parameters: database TAIR10 (release 2010/12/14, 35386 sequences); enzymatic specificity: trypsin permitting two allowed missed cleavages; fixed modification of cysteine residues (Methylthio(C)); possible variable modifications of phosphorylation on S, T and Y residues; 5 ppm tolerance on precursor masses and 0.5 Da tolerance on fragment ions. The results were filtered based on Mascot scores and MD-scores.

**Bimolecular fluorescence complementation (BiFC)**

To obtain the expression vectors, coding sequences of candidate genes and MPK4 (kindly provided by J. Colcombet) were cloned in fusion with the N- and C-terminal parts of YFP, either as N- or C-terminal fusions, under the control of the cauliflower mosaic virus 35S (CaMV-35S) promoter in the pBIFC1, 2, 3 and 4 vectors [6]. Appropriate positive and negative controls were carried out for all combinations. Recombined vectors were individually transformed in *Agrobacterium tumefaciens* C58C1 strain by electroporation. Agrobacterium cultures from glycerol stocks were inoculated in 10 ml of LB medium with appropriate antibiotics and incubated for 24 h at 28°C with agitation. Each culture was pelleted and resuspended in infiltration buffer (10 mM MgCl_2_, 10 mM MES pH 5.7, 150 µM acetosyringone) to an OD_600_ of 1.5 and kept in the dark for 3 h. The P19 viral suppressor of gene silencing was co-expressed with each combination to prevent silencing of transiently expressed proteins [7]. 500 µl of each bacterial culture was mixed before infiltration. For fluorescence complementation, all eight possible combinations between a candidate gene and a MAPK were agro-infiltrated into 3-week-old *Nicotiana benthamiana* leaves. After 3 days, an upright LSM 710 Zeiss confocal microscope with a 20X objective (Plan-Apochromat, NA 1.0) was used to visualise fluorescence. All images were acquired by the use of Argon laser with 514-nm excitation.

**Tandem affinity purification (TAP) coupled to mass spectrometry analysis**

The TAP approach and mass spectrometry analysis were performed as described by Bigeard et al, 2014 [8], with the following modifications. For protein precipitation, after the two steps of purification, we used a standard TCA/acetone precipitation method instead of the methanol precipitation method. Then, the samples were analyzed with a Q Exactive mass spectrometer (Thermo Fisher) instead of the LTQ-Orbitrap XL instrument (Thermo electron). The acquired MS/MS data were interpreted by the use of the Mascot server v2.2.07 (matrixscience.com) while specifying the following parameters: database TAIR10 (release 2010/12/14, 35386 sequences): enzymatic specificity: tryptic with three allowed missed cleavages; fixed modification of cysteine residues (Methylthio(C)); possible phosphorylation of S, T and Y residues, oxidation of Met and acetylation of protein N-termini; 5 ppm tolerance on precursor masses and 0.025 ppm tolerance on fragment ionsRelative quantification of peptides between the different analyzed samples was performed by the use of the software Proline (<http://www.profiproteomics.fr/>). The maximum MS intensity of the LC elution peaks of peptides was determined by the program. Then the abundance of GTL1 in the sample was determined by summing the MS signals quantified on its specific peptides. The normalization was based on MPK4 protein abundance that was set to 1 in each biological replicate, before or after 15 min flg22 treatment.

**DAB staining**

Stainings were conducted by the use of 14 day-old seedlings grown under sterile conditions on half MS medium, in accordance with [9].

**ROS burst assay**

ROS burst assay was performed as described by [10].

1. Rayapuram, N., et al., *Quantitative phosphoproteomic analysis reveals shared and specific targets of Arabidopsis MPK3, MPK4 and MPK6.* Mol Cell Proteomics, 2017.

2. Tian, T., et al., *agriGO v2.0: a GO analysis toolkit for the agricultural community, 2017 update.* Nucleic Acids Res, 2017.

3. Nallamsetty, S., et al., *Gateway vectors for the production of combinatorially-tagged His6-MBP fusion proteins in the cytoplasm and periplasm of Escherichia coli.* Protein Sci, 2005. **14**(12): p. 2964-71.

4. Grefen, C., et al., *A ubiquitin-10 promoter-based vector set for fluorescent protein tagging facilitates temporal stability and native protein distribution in transient and stable expression studies.* Plant J, 2010. **64**(2): p. 355-65.

5. Jelenska, J., J.A. van Hal, and J.T. Greenberg, *Pseudomonas syringae hijacks plant stress chaperone machinery for virulence.* Proc Natl Acad Sci U S A, 2010. **107**(29): p. 13177-82.

6. Azimzadeh, J., et al., *Arabidopsis TONNEAU1 proteins are essential for preprophase band formation and interact with centrin.* Plant Cell, 2008. **20**(8): p. 2146-59.

7. Voinnet, O., et al., *An enhanced transient expression system in plants based on suppression of gene silencing by the p19 protein of tomato bushy stunt virus.* Plant J, 2003. **33**(5): p. 949-56.

8. Bigeard, J., et al., *Protein complexes characterization in Arabidopsis thaliana by tandem affinity purification coupled to mass spectrometry analysis.* Methods Mol Biol, 2014. **1171**: p. 237-50.

9. Genot, B., et al., *Constitutively active Arabidopsis MAP Kinase 3 triggers defense responses involving salicylic acid and SUMM2 resistance protein.* Plant Physiol, 2017.

10. Smith, J.M. and A. Heese, *Rapid bioassay to measure early reactive oxygen species production in Arabidopsis leave tissue in response to living Pseudomonas syringae.* Plant Methods, 2014. **10**(1): p. 6.
